# Associations between exploratory dietary patterns and incident type 2 diabetes: a federated meta-analysis of individual participant data from 25 cohort studies

**DOI:** 10.1007/s00394-022-02909-9

**Published:** 2022-06-01

**Authors:** Franziska Jannasch, Stefan Dietrich, Tom R. P. Bishop, Matthew Pearce, Anouar Fanidi, Gráinne O’Donoghue, Donal O’Gorman, Pedro Marques-Vidal, Peter Vollenweider, Maira Bes-Rastrollo, Liisa Byberg, Alicja Wolk, Maryam Hashemian, Reza Malekzadeh, Hossein Poustchi, Vivian C. Luft, Sheila M. Alvim de Matos, Jihye Kim, Mi Kyung Kim, Yeonjung Kim, Dalia Stern, Martin Lajous, Dianna J. Magliano, Jonathan E. Shaw, Tasnime Akbaraly, Mika Kivimaki, Gertraud Maskarinec, Loïc Le Marchand, Miguel Ángel Martínez-González, Sabita S. Soedamah-Muthu, EPIC-InterAct Consortium, Nicholas J. Wareham, Nita G. Forouhi, Matthias B. Schulze

**Affiliations:** 1 Department of Molecular Epidemiology, German Institute of Human Nutrition Potsdam-Rehbruecke, Nuthetal, Germany; 2 NutriAct Competence Cluster Nutrition Research Potsdam-Berlin, Nuthetal, Germany; 3 German Center for Diabetes Research, Munich-Neuherberg, Germany; 4 Department of Food Safety, German Federal Institute for Risk Assessment (BfR), Berlin, Germany; 5 MRC Epidemiology Unit, School of Clinical Medicine, Institute of Metabolic Science, University of Cambridge, Cambridge Biomedical Campus, Cambridge, CB2 0QQ UK; 6 School of Public Health, Physiotherapy and Sports Science, University College Dublin, Dublin, Ireland; 7 School of Health and Human Performance, Dublin City University, Dublin, Ireland; 8 Department of Medicine, Internal Medicine, Lausanne University Hospital and University of Lausanne, Office BH10-642, Rue du Bugnon 46, 1011 Lausanne, Switzerland; 9 Department of Preventive Medicine and Public Health, University of Navarra, Pamplona, Spain; 10 CIBERobn, Instituto de Salud Carlos III, Madrid, Spain; 11 Navarra Institute for Health Research (IdiSNA), Pamplona, Spain; 12 Department of Surgical Sciences, Medical Epidemiology, Uppsala University, Uppsala, Sweden; 13 Institute of Environmental Medicine, Karolinska Institutet, Stockholm, Sweden; 14 Digestive Disease Research Center, Digestive Disease Research Institute, Tehran University of Medical Sciences, Tehran, Iran; 15 Biology Department, School of Arts and Sciences, Utica College, Utica, NY USA; 16 Liver and Pancreatobiliary Diseases Research Center, Digestive Diseases Research Institute, Tehran University of Medical Sciences, Tehran, Iran; 17 Faculdade de Medicina, Universidade Federal do Rio Grande do Sul (UFRGS), Porto Alegre, RS Brazil; 18 Institute of Collective Health, Federal University of Bahia, Salvador, Bahia Brazil; 19 Department of Preventive Medicine, College of Medicine, Hanyang University, Seoul, South Korea; 20 Division of Health and Nutrition Survey and Analysis, Korea Disease Control Prevention Agency, Seoul, South Korea; 21 CONACyT-Center for Research on Population Health, National Institute of Public Health, Cuernavaca, Morelos Mexico; 22 Baker Heart and Diabetes Institute, 75 Commercial Road, Melbourne, VIC 3004 Australia; 23 Inserm U 1018, Université Paris-Saclay, UVSQ, Villejuif, Maison des Sciences de l’Homme – SUD, Montpellier, France; 24 Department of Epidemiology and Public Health, University College London, London, UK; 25 University of Hawaii Cancer Center, Honolulu, HI USA; 26 Department of Nutrition, Harvard T.H. Chan School of Public Health, 665 Huntington Avenue, Boston, MA USA; 27 Center of Research On Psychological and Somatic Disorders (CORPS), Department of Medical and Clinical Psychology, Tilburg University, PO Box 90153, 5000 LE Tilburg, The Netherlands; 28 Institute for Food, Nutrition and Health, University of Reading, Reading, RG6 6AR UK

**Keywords:** Dietary patterns, Exploratory, Type 2 diabetes mellitus, Federated meta-analysis

## Abstract

**Purpose:**

In several studies, exploratory dietary patterns (DP), derived by principal component analysis, were inversely or positively associated with incident type 2 diabetes (T2D). However, findings remained study-specific, inconsistent and rarely replicated. This study aimed to investigate the associations between DPs and T2D in multiple cohorts across the world.

**Methods:**

This federated meta-analysis of individual participant data was based on 25 prospective cohort studies from 5 continents including a total of 390,664 participants with a follow-up for T2D (3.8–25.0 years). After data harmonization across cohorts we evaluated 15 previously identified T2D-related DPs for association with incident T2D estimating pooled incidence rate ratios (IRR) and confidence intervals (CI) by Piecewise Poisson regression and random-effects meta-analysis.

**Results:**

29,386 participants developed T2D during follow-up. Five DPs, characterized by higher intake of red meat, processed meat, French fries and refined grains, were associated with higher incidence of T2D. The strongest association was observed for a DP comprising these food groups besides others (IRR_pooled_ per 1 SD = 1.104, 95% CI 1.059–1.151). Although heterogeneity was present (*I*^2^ = 85%), IRR exceeded 1 in 18 of the 20 meta-analyzed studies. Original DPs associated with lower T2D risk were not confirmed. Instead, a healthy DP (HDP1) was associated with higher T2D risk (IRR_pooled_ per 1 SD = 1.057, 95% CI 1.027–1.088).

**Conclusion:**

Our findings from various cohorts revealed positive associations for several DPs, characterized by higher intake of red meat, processed meat, French fries and refined grains, adding to the evidence-base that links DPs to higher T2D risk. However, no inverse DP–T2D associations were confirmed.

**Supplementary Information:**

The online version contains supplementary material available at 10.1007/s00394-022-02909-9.

## Introduction

A large number of prospective studies have evaluated dietary patterns (DP) in relation to the risk of developing type 2 diabetes mellitus (T2D) [1]. While evidence for a priori, also called hypothesis-driven, DPs like the Mediterranean diet is convincing [2], evidence for DPs derived by exploratory methods using the data at hand is inconsistent [1]. Several studies reported associations of study-specific exploratory DPs with higher T2D risk, some of them labelled “Western” [3–11]. Such DPs frequently included red meat [3–7, 9, 10], refined grains [3–10], sugary drinks [3, 7, 8, 10] or French fries [3–6, 8–10]. However, the composition of these exploratory DPs still differs in other food groups (FG) besides those mutual ones (1) and the food groups per se can comprise different food items based on the study specific assessment and dietary habits. In addition, similarly labelled DPs (e.g. “Western”) showed heterogeneous associations with T2D risk [1, 12]. Thus, the exploratory nature of DPs results in study-specific observations rather than generalizable findings. So far, little effort has been made to assess the actual generalizability of DP–T2D associations. This limits the accumulation of consistent evidence from cohort studies on DP associations with T2D—thus, evidence from exploratory DPs to inform dietary recommendations has been sparse.

A solution to overcome the limitation of study-specific findings is to replicate the association of DPs with T2D in independent populations. So far, only one study investigated the generalizability of T2D-associations with DPs derived by principal component analysis (PCA). However, this study was restricted to European populations participating in the EPIC-InterAct consortium with the aim to replicate only those T2D-associated DPs which were derived in country-specific analyses within this consortium [13]. In addition to PCA, patterns derived by reduced rank regression, were also replicated [14–16]. The main principle for those replication approaches is the reconstruction of pattern variables based on the reported pattern structure. In this context, it has been proposed to derive so-called simplified DP variables to construct less population-dependent DP variables with a content approximately similar to that of original exploratory DPs. It has been shown that the DP variables, calculated with this method, correlated highly with the original DP and reflected variation in intake of individual components well [14, 16, 17]. Hence, this approach seems well suited to replicate study-specific associations of exploratory DPs in independent study populations. To date, however, this method has not been used to examine exploratory DPs in relation to T2D across populations from different continents of the world.

To overcome the research gap of investigating the generalizability of DP–T2D associations using the approach of simplified DPs, the present study aimed 1) to investigate the association of previously reported T2D-associated DPs [1] with incident T2D and 2) to evaluate, if two DPs of overlapping FGs (“mainly healthy” and “mainly unhealthy”), also previously identified in the same systematic review [1], are associated with incident T2D. For this purpose, the InterConnect collaboration project offers a well-suited research platform for federated meta-analyses of harmonized individual level study data from 25 cohorts [18–33] across different continents and adjusting for a common set of potential confounders across studies [34–36]. As another advantage, this approach allowed the inclusion of cohorts that have relevant data, but never published on the topic before.

## Methods

### Study populations

InterConnect was an EU-FP7 funded project which aimed to optimise the use of existing data by enabling cross-cohort analyses within consortia without pooling of data at a central location ("http://www.interconnect-diabetes.eu/") [34]. For the current study, the InterConnect Data Discovery registry (http://www.interconnect-diabetes.eu/data-discovery/) and literature was screened to identify cohorts with suitable data like study populations representing the general population without prevalent T2D, dietary intake information (amount, frequency), incident T2D as outcome (self-report, objective measures), and information on the covariates age, sex, smoking, body mass index (BMI), waist circumference or waist-hip ratio, physical activity, alcohol consumption, education or occupation, family history of diabetes, other health exposures (cardiovascular diseases, history of previous illness). Of 103 identified cohorts, 25 collaborating cohorts (Table S1) contributed data to this project [18–33, 37]. The Zutphen Elderly study also contributed data, but was excluded due to a too low number of cases [37]. Other reasons for non-participation (Fig. S1) were failed contact (*n* = 46), no interest in research question (*n* = 10), insufficient data (*n* = 15) or no study capacity (*n* = 6). The collaborating cohorts [18–32, 38] included 13 cohorts from Europe, eight from the Americas (North and South America), three from Western Pacific (Australia, Republic of Korea), and one from the Eastern Mediterranean (Iran). All cohorts obtained ethical review board approval at the host institution and informed consent from participants.

### Dietary assessment and construction of dietary patterns

Dietary intake was assessed by food frequency questionnaires (FFQ) in most cohorts, by dietary history interview and a 24-h recall in one cohort each (Table S1). For the present study food intake encoded in g/day was used. Some cohorts provided only standard portion sizes and frequency of consumed food items, which were converted into g/day. For some US cohorts, where information on portion size was not available, variable-specific standard portion sizes sourced from the United States Department of Agriculture [39] were used.

The dietary data of all cohorts were then harmonized to form a set of food groups. For this purpose, the FGs used in the published DPs associated with T2D risk were compared. Based on this, a set of FGs was defined to be used across all published DPs (Tables 1, S2 and S3). If for a specific food item, which was used in the original DP, no intake information was available in other included studies, it was omitted. Then the respective study-specific food items were added in each InterConnect cohort to form the corresponding harmonized FG (Excel Table S6). Subsequently, DPs were constructed based on the harmonized FGs. The structure of DPs was defined based on the findings of our previous systematic review [1], thus reflecting a) DPs found to be significantly associated with T2D risk in at least one cohort study (13 individual DPs) and b) two DPs reflecting DPs with overlapping food composition: the DP reflecting the overlap of “mainly healthy” food groups was composed of fruits, vegetables, legumes, poultry and fish, while the DP of “ mainly unhealthy” food groups was composed of refined grains, French fries, red meat, processed meat, high-fat dairy products and eggs. Thus, 15 DPs in total were constructed. To calculate individual DP scores for study participants, the approach of simplified DPs [17] was used. In PCA-derived DPs, all food groups contribute with a respective factor loading to the overall pattern structure. The simplification approach considers only those FGs with strong contribution to the respective DP (factor loading (FL) ≥ 0.2) in the original DPs. Details of which FGs were combined to calculate the respective simplified DP scores are shown in Tables 1, S2 and S3. These FGs were standardized according to the distribution in each participating study, respectively. Then, simplified DP scores were calculated by summing up the selected FGs without any weighting (in original DP the respective FL is the weighting) and by also considering negative algebraic signs for those FGs with negative FL from the original publication. Finally, study-specific simplified DP scores were also standardized to allow meta-analysis across cohorts [17].

**Table 1:** Risk estimates for T2D from the original studies, where DPs were derived in and composition of simplified pattern variables used for the analyses in InterConnect

DP	Published by	Published risk estimate^a^ for T2D	Harmonized food groups used in InterConnect to replicate published dietary pattern^b^
HDP 1	Montonen, 2005 [4] Finnish Mobile Clinic Health Examination Survey	0.72 (0.53–0.97)	**Vegetables + Fruits** + Poultry + Eggs + Red meat—Whole milk + Nuts + High fat dairy + low-medium fat dairy + Margarine + Fish
HDP 2	Erber, 2010 [6] Multiethnic cohort	Men: 0.86 (0.77–0.95) Women: 1.02 (0.91–1.14)	**Vegetables + Fruits**
HDP 3	Erber, 2010 [6] Multiethnic cohort	Men: 0.92 (0.83–1.02) Women: 0.85 (0.76–0.96)	**(Whole Milk, High-fat dairy, medium/low-fat dairy) + Fruits** + (Cheese, low-fat cheese)
HDP 4	Odegaard, 2011 [11] Singapore Chinese Health Study	Never smoker: 0.77 (0.65–0.92) Smoker: 1.17 (0.91–1.51)	**Vegetables + Potatoes + Legumes/soy** + Fruits + Fish + Poultry
HDP 5	Yu, 2011 [7] Hong Kong Dietary Survey	0.76 (0.58–0.99)	**(Fish, Shellfish) + Fruits + Vegetables** + Legumes/Soy + Nuts
HDP 6	Morimoto, 2012 [53] Rural Japanese population study	0.78 (0.61–0.95)	**Vegetables + Potatoes without fries + Fruits** + Legumes/Soy + (Butter, margarine, mayonnaise) + (Fish, Shellfish)
UDP 1	van Dam, 2002 [3] Health Professionals Follow-up Study	Men: 1.59 (1.32–1.93)	**Red meat + Processed meat + (refined grains, pasta, rice) + French fries + (whole milk, high fat-diary, cheese, ice cream) + (cake, confect) + eggs + (Sugar & Confectionary, condiment sauces)** + Sugary soft drinks + Butter + Mayonnaise + Potatoes + Margarine + Pizza + Coffee + Nuts
UDP 2	Montonen, 2005 [4] Finnish Mobile Clinic Health Examination Survey	1.49 (1.11–2.00)	**Butter + (Potatoes, French fries) + Whole milk + (Red meat, Offals) + Sugar & Confectionary + Whole grain bread + (Rice, whole grain cereals) + Processed meat** + (Pasta, refined grains) + Eggs + Fish + Shellfish + (Nuts, Legumes, Soy)
UDP 3	Hodge, 2007 [5] Melbourne Collaborative Cohort Study	1.65 (1.03–2.63)	**Red meat** + French fries + Eggs + Fish + Processed meat + Refined grain bread + Poultry + Rice
UDP 4	Erber, 2010 [6] Multiethnic cohort	Men: 1.40 (1.23–1.60) Women: 1.22 (1.06–1.40)	**(Butter, margarine) + (Red meat, offals) + Processed meat + (Potatoes, French fries) + Refined grains + Eggs + (Cheese, low-fat cheese)**
UDP 5	Yu, 2011 [7] Hong Kong Dietary Survey	1.39 (1.04–1.84)	**(Red meat, processed meat) + (High fat-diary, medium/low-fat diary, cheese, low-fat cheese, ice)** + Sugar and Confectionary + Eggs + (Refined grains, pasta, white rice) + Sugary soft drinks + Poultry + Offals
UDP 6	Bauer, 2013 [8] EPIC-Netherlands	1.70 (1.31–2.20)	**Sugary soft drinks + Low sugary soft drinks + French fries + Pizza – Fruits—medium/low-fat dairy—(Whole grain bread, whole grain cereals) + refined grain bread—Legumes—Cakes—Vegetables**
UDP 7	Schoenaker, 2013 [9] Australian Longitudinal Study on Womens Health	Women: 1.73 (1.12–2.67)	(Whole milk, cheese) + Refined grain bread + Red meat + Processed meat + Pizza + French fries + (Cakes, ice cream, Confectionary)

*CI* confidence interval, *DP* dietary pattern, *HDP* healthy dietary pattern, *UDP* unhealthy dietary pattern

^a^All published risk estimates are shown for the comparison of extreme intakes, except for Yu et al. 2011, where it is shown per 1 standard deviation increase. *T2D* type 2 diabetes; ^b^Harmonized food groups in bold represent food groups with published factor loadings > 0.4 and in bold represent food groups with published factor loadings 0.4–0.2 in the original publication. Food groups in brackets represent harmonized food groups that were combined for simplified pattern variable calculation to replicate the food group as used in the original publication

### Ascertainment of incident T2D

To minimize potential variations due to varying diagnosis criteria of T2D incidence across cohorts, two harmonized outcomes were defined [40]. As primary outcome, clinically incident T2D was defined when any one or more of the following criteria were fulfilled: (1) ascertained by linkage to a registry or medical record; (2) confirmed antidiabetic medication usage; (3) self-report of physician diagnosis or antidiabetic medication, verified by any of the following: (a) at least one additional source from 1 or 2 above, (b) biochemical measurement (glucose or HbA1c), (c) a validation study with high concordance. As secondary outcome with less strict criteria, we defined incident T2D, when any of the following criteria were fulfilled: (1) ascertained by linkage to a registry or medical record; (2) confirmed antidiabetic medication usage; (3) self-report of physician diagnosis or antidiabetic medication or (4) biochemical measurement (glucose or HbA1c).

### Assessment of covariates

We defined a set of potential confounders to be used in analyses based on: (1) frequent usage in the studies of the 13 published T2D-associated DPs and (2) availability across all participating InterConnect cohorts (Table S4). The final set of confounders included: age at baseline (years), sex, body mass index (BMI) (kg/m^2^), physical activity (PA, cohort specific items were used), education (cohort specific items were used), smoking (never, former, current smoker), alcohol consumption (g/day), hypertension (yes/no), and energy intake (kcal/day). The recorded data of confounders of the respective InterConnect cohorts were used and harmonized across all cohorts, if possible (Table S5). All cohorts provided age in years, BMI in kg/m^2^, hypertension as yes or no. Smoking was harmonized as never, former, and current smoker, energy intake into kcal/day and alcohol into g/day. In the Golestan Cohort Study from Iran alcohol consumption was used as never or ever drinker. Study-specific coding was used for PA and education because harmonization was not feasible due to extensive differences in codes (Table S5).

### Statistical analysis

All analyses were conducted using R within the DataSHIELD federated meta-analysis programming library [35]. For analysis, participants with the following criteria were excluded: T2D, myocardial infarction, stroke or cancer at baseline to avoid reverse causation, extreme energy intake (men < 800 kcal or > 4200 kcal, women < 500 kcal or > 3500 kcal), missing follow up time, missing confounders, and more than 10% missing food items. In total, 46.9% of the participants of the InterConnect cohorts were excluded (Table 2). Baseline characteristics were calculated stratified by cohorts. Normally distributed variables were presented as mean and standard deviation (SD), not normally distributed as median and interquartile range (IQR), and categorical variables as relative percentages.

**Table 2:** Characteristics of analyzed data^a^ of the participating 25 InterConnect cohorts

Study (country)	Analysis sample (*n*)	Primary T2D cases (*n*)	Secondary T2D cases (*n*)	Exclusions (% of total)	Follow-up time (years)	Age (years)	Wo-men (%)	BMI (kg/m^2^)	Energy Intake (kcal)	Prevalent hypertension (%)	Never smoker (%)	Former smoker (%)	Current smoker (%)	Alcohol intake (g/day)
ARIC (USA) [18]	8750	656	1824	44.6%	16.7 (8.9–23.7)	53.6 (5.6)	56.5	27.2 (5.0)	1634 (593)	29.8	45.0	32.9	22.1	6.2 (13.1)
AusDiab (Australia) [30]	5932	183	359	47.3%	11.7 (5.1–12.2)	49.7 (12.3)	56.1	26.6 (4.7)	1909 (652)	26.0	59.6	28.6	11.8	14.7 (18.9)
CARDIA (USA) [19]	3737	186	372	26.7%	25.0 (19.0–25.0)	24.9 (3.6)	58.0	24.3 (4.7)	2336 (804)	8.9	59.1	14.0	27.0	9.1 (14.2)
CoLaus (Switzerland) [25]	3697	200	248	27.0%	10.7 (10.5–10.9)	56.8 (10.3)	56.2	25.8 (4.3)	1816 (622)	30.5	42.3	36.9	20.7	6.2 (7.8)
COSM (Sweden) [26]	24,278	2613	2647	47.1%	18.0 (18.0–18.0)	58.5 (9.0)	0	25.6 (3.1)	2622 (659)	18.3	36.2	39.9	24.0	15.6 (19.1)
ELSA-Brasil (Brasil) [20]	10,647	308	935	29.5%	3.8 (3.5–4.1)	51.2 (8.8)	56.5	26.7 (4.6)	2409 (692)	30.8	59.1	28.6	12.3	6.5 (12.9)
Golestan (Iran) [24]	9546	633	1091	80.9%	4.2 (3.6–5.6)	51.1 (7.8)	52.0	26.8 (5.3)	2196 (554)	15.4	82.4	3.7	13.9	0^b^
InterAct Denmark [27]	3222	1605	1605	20.2%	10.6 (6.4–11.6)	56.9 (4.4)	46.7	27.2 (4.5)	2227 (582)	23.6	32.4	31.7	35.9	21.5 (22.8)
InterAct France [27]	689	224	224	20.5%	9.3 (7.4–10.5)	56.7 (6.6)	100	24.6 (4.8)	2156 (514)	19.0	67.6	22.9	9.4	10.9 (14.6)
InterAct Germany [27]	3167	1361	1361	11.5%	9.6 (4.9–11.3)	52.1 (8.3)	50.6	27.6 (4.8)	2071 (607)	41.0	43.6	35.2	21.2	16.9 (20.9)
InterAct Italy [27]	3027	1229	1,229	10.8%	10.9 (6.9–12.7)	51.4 (7.7)	65.4	27.4 (4.8)	2278 (628)	26.7	45.2	26.8	28.0	13.3 (18.4)
InterAct Netherlands [27]	1811	624	624	20.9%	11.2 (6.7–12.7)	54.3 (9.7)	84.5	26.6 (4.5)	1925 (519)	25.8	41.1	32.6	26.3	9.3 (13.6)
InterAct Spain [27]	5480	2296	2296	6.9%	12.5 (9.0–13.6)	50.3 (7.8)	56.6	29.3 (4.6)	2171 (659)	24.4	53.7	17.2	29.1	15.5 (24.2)
InterAct Sweden [27]	4598	2080	2080	14.9%	12.0 (9.4–13.6)	54.4 (9.7)	51.4	26.7 (4.6)	2111 (635)	23.7	43.9	29.7	26.4	7.6 (10.5)
InterAct UK [27]	1647	507	507	29.1%	10.7 (6.6–12.3)	57.7 (10.5)	53.9	26.9 (4.5)	2024 (568)	17.6	48.0	38.2	13.8	8.5 (12.1)
KoGES ASAS (Korea) [31]	5085	–	769	49.6%	7.7 (5.1–7.9)	50.5 (8.6)	51.7	24.5 (3.0)	1837 (510)	11.9	59.7	16.1	24.2	9.8 (21.7)
KoGES CAVAS (Korea) [31]	6620	–	290	69.5%	4.3 (3.3–5.5)	61.0 (9.9)	63.7	24.2 (3.1)	1518 (456)	19.2	69.6	15.3	15.1	13.0 (38.5)
MEC (USA) [32]	121,329	6387	6387	43.8%	17.0 (16.1–17.6)	58.5 (8.8)	42.6	25.9 (4.6)	1995 (705)	32.7	47.2	37.2	15.6	8.0 (18.9)
MESA (USA) [33]	4507	205	632	33.9%	9.0 (7.4–10.0)	61.2 (10.2)	54	28.0 (5.2)	1540 (673)	34.8	51.6	35.8	12.6	5.5 (12.1)
MTC (Mexico) [21]	52,434	–	1706	54.5%	6.0 (6.0–6.0)	41.8 (7.5)	100	27.1 (4.6)	1821 (616)	12.7	78.7	12.1	9.2	0.7 (1.7)
PRHHP (Puerto Rico) [22]	6807	–	806	22.6%	5.0 (5.0–5.0)	54.0 (6.5)	0	25.0 (3.9)	2351 (718)	27.0	33.6	22.6	43.8	9.8 (27.9)
SMC (Sweden) [26]	22,213	1804	1844	63.8%	18.0 (18.0–18.0)	59.8 (8.3)	100	24.8 (3.7)	1748 (474)	17.8	48.8	26.3	24.9	6.9 (8.9)
SUN (Spain) [28]	9371	–	173	42.8%	10.2 (6.2–12.8)	39.4 (12.0)	57.6	23.9 (3.6)	2344 (639)	7.8	49.8	46.3	3.8	8.6 (11.9)
Whitehall II (UK) [29]	3847	447	558	62.7%	16.2 (15.5–6.6)	49.5 (5.9)	28.9	25.2 (3.7)	2092 (585)	17.3	47.5	39.9	12.6	17.9 (21.1)
WHI (USA) [23]	68,223	5733	5960	27.2%	11.9 (8.1–13.7)	63.1 (7.3)	100	26.9 (5.5)	1528 (553)	30.0	51.5	42.5	6.0	5.7 (11)
Total	390,664	29,386	36,527	46.9%	–	–	–	–	–	–	–	–	–	–

^a^Data are reported as percentage, mean ± SD for normally distributed or median (IQR) for non-normally distributed variables. Shown are analyzed data after application of exclusion criteria

^b^In Golestan Cohort study only 4% of the participants ever consumed alcohol

Incidence rate ratios (IRRs) and 95% confidence intervals (CI) were estimated to test for the associations between 1 standard deviation (SD) increase in DP scores and incident T2D in each cohort separately, using Piecewise Poisson regression adjusted for age, sex, BMI, PA, education, smoking, alcohol consumption, hypertension and energy intake. The Piecewise Poisson regression is available in the DataSHIELD library and has been shown to represent a close approximation to the Cox Proportional Hazards regression [41]. For the European Prospective Investigation into Cancer and Nutrition (EPIC)-InterAct cohorts a weighting was applied that is analogous to Prentice weighting (weights of 1 for all cases and weights of $\frac{\#\mathrm{ non}-\mathrm{cases in whole cohort}}{\#\mathrm{ non}-\mathrm{cases in subcohort}}$ for non-cases) to account for the case-cohort design in survival analyses, when using the piecewise Poisson method [42].

Pooled IRR were estimated using random-effects meta-analysis models and were visualized with forest plots. Heterogeneity was assessed using *I*^2^, *p* value of chi-square test and tau^2^ statistic. For each DP a statistical model for the primary and the secondary outcome was calculated. For sensitivity analysis we calculated a second set of the 13 DPs by considering only FGs with FL ≥ 0.4 in the original publication to identify those strongly contributing to the DP. Moreover, a sensitivity analysis with exclusion of certain component FGs was conducted to estimate if few FGs were mainly driving the association from the UDP3, which showed the strongest association with T2D. To account for characteristics potentially explaining heterogeneity between the cohorts, meta-regressions were calculated with the pooled IRR as dependent variable and age, BMI, follow-up time and region as the independent variables. For this, the metareg function within the metafor package (version 3.02) in R was used.

## Results

In the present analysis, data from 390,664 participants across 25 cohorts with a median follow-up time ranging from 3.8 to 25.0 years were included (Table 2). Four cohorts included only women (EPIC-InterAct-France, Mexican Teachers' Cohort (MTC), Swedish Mammography Cohort (SMC), Women's Health Initiative Observational Study (WHI-OS)) and two only men (Cohort of Swedish Men (COSM), Puerto Rico Heart Health Program (PRPHH)). Participants from Coronary Artery Risk Development in Young Adults (CARDIA) study, MTC and Seguimiento University of Navarra (SUN) cohort were of younger age (24.9–41.8 years), whereas participants from other cohorts were older (49.5–63.1 years). The mean BMI ranged from 23.9 kg/m^2^ in SUN to 29.3 kg/m^2^ in EPIC-InterAct-Spain. During follow-up, 29,386 clinically incident cases of T2D were recorded for the primary outcome and 36,527 incident cases for the secondary outcome.

The dietary intake of harmonized FGs showed marked differences between the cohorts **(**Excel Supplemental Table). For example, reported median fruit intake was highest in MTC (321.7 g/day) and about three times higher than median intake in the cohorts with lowest fruit intake like CARDIA (94.9 g/day) and EPIC-InterAct-Germany (91.4 g/day). Particularly high intakes compared to other cohorts were observed for vegetables in SUN Study (391 g/day), legumes and soy (but mostly beans) in Brazilian Longitudinal Study of Adult Health (ELSA-Brasil) (151.0 g /day), refined grains in Golestan (365.0 g/day), whole grains in COSM (127.0 g/day) and EPIC-InterAct-Germany (120.3 g/day), and sugary drinks in CARDIA (244.9 g/day).

### Healthy dietary patterns and risk of T2D

**Fig. 1:**
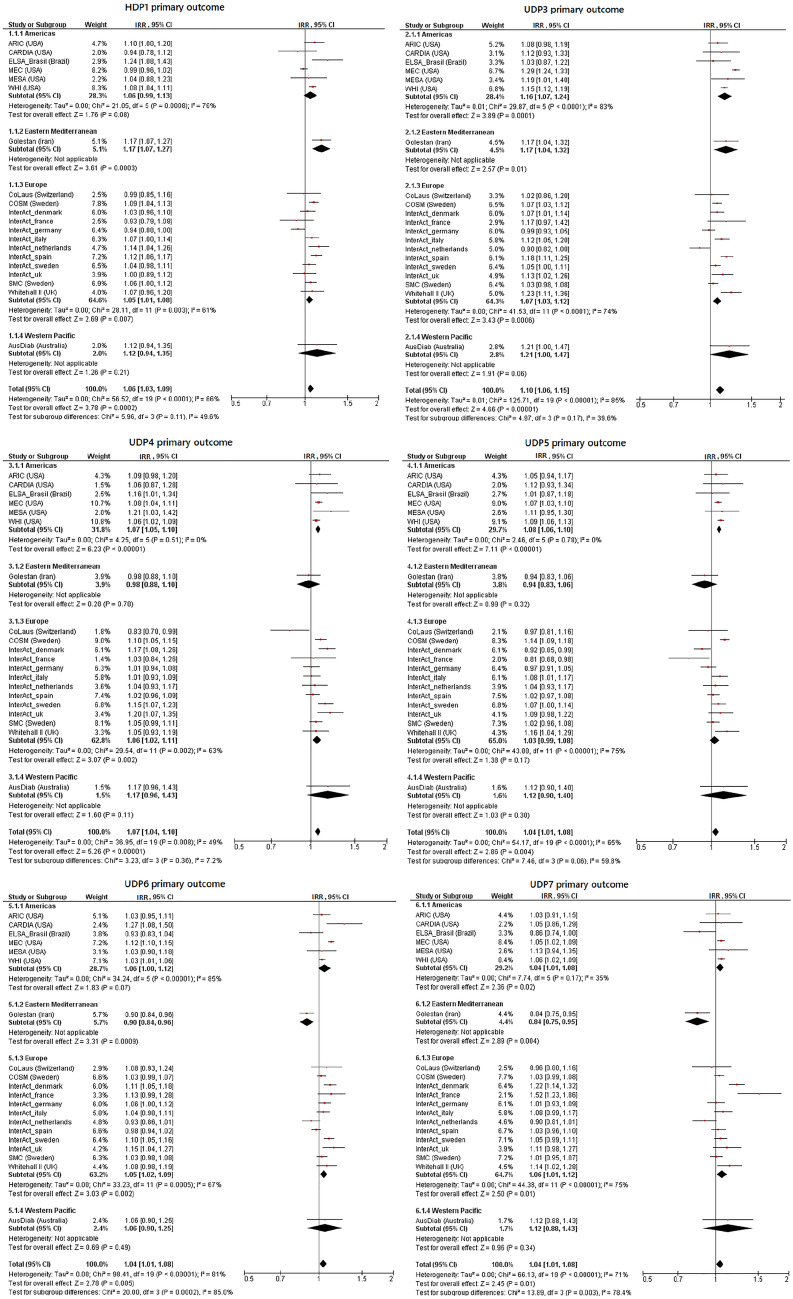
Incidence rate ratios and 95% confidence intervals for the association between replicated dietary pattern variables and incident type 2 diabetes. Shown are results for the primary outcome definition and harmonized food groups with published factor loadings > 0.2 by subgroups of region. Associations are adjusted for age, sex, BMI, physical activity, education, smoking, alcohol consumption, total energy intake and hypertension. *CI* confidence intervals, *IRR* incidence rate ratios, *HDP* healthy dietary pattern, *UDP* unhealthy dietary patterns

**Fig. 2:**
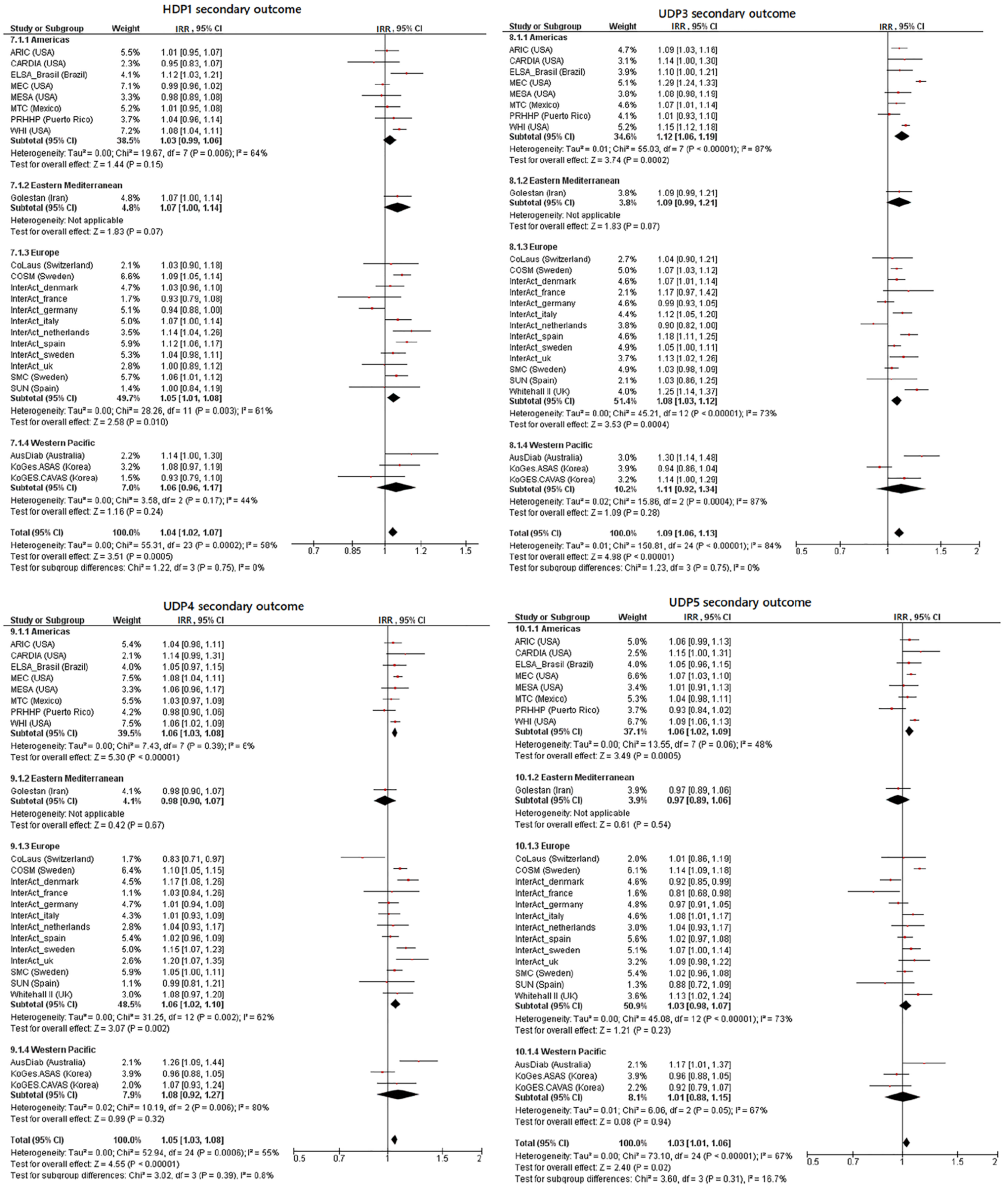

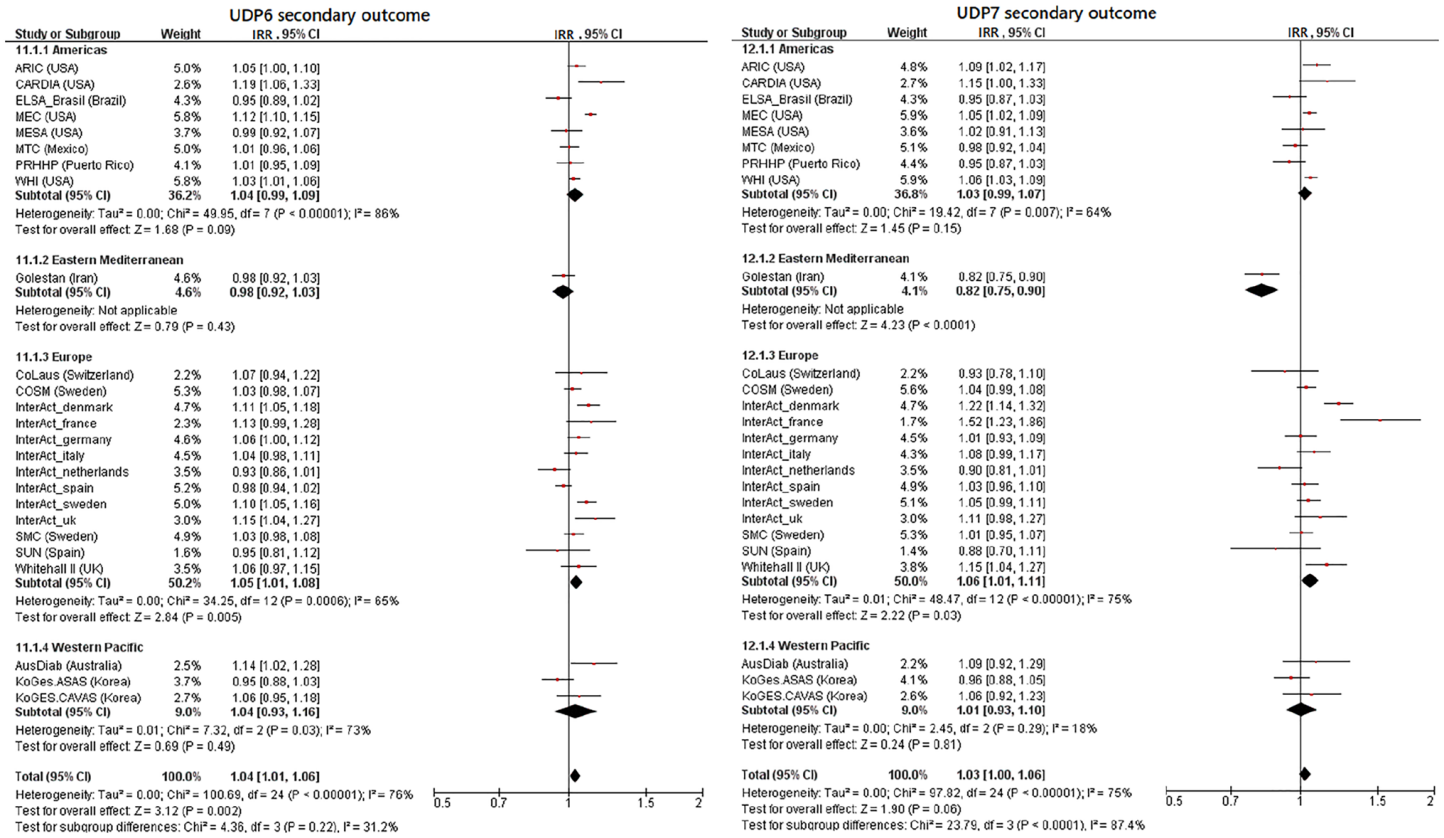
Incidence rate ratios and 95% confidence intervals for the association between replicated dietary pattern variables and incident type 2 diabetes. Shown are results for the secondary outcome definition and harmonized food groups with published factor loadings > 0.2 by subgroups of region. Associations are adjusted for age, sex, BMI, physical activity, education, smoking, alcohol consumption, total energy intake and hypertension. *CI* confidence intervals, *IRR* incidence rate ratios, *HDP* healthy dietary pattern, *UDP* unhealthy dietary patterns

None of the HDPs (Table 3, Figs. 1, 2, Supplemental Table 6, Supplemental Figs. 2–5) were robustly associated with a reduced risk of T2D. This was the case for the two outcome definitions and for the two versions of each HDP constructed using different cut-offs of FL to define component FGs. HDP1 was significantly associated with a higher T2D risk (primary outcome: pooled IRR per SD = 1.057, 95% CI 1.027–1.088; secondary outcome: IRR per SD = 1.042, 95% CI 1.018–1.065, Table 3). This DP contains vegetables, fruits, margarine, nuts, poultry, eggs, fish, red meat, whole milk, high fat dairy and low-medium fat dairy. However, this association was absent in sensitivity analysis, when only FGs with published absolute FL ≥ 0.4 (vegetables and fruits, Table 2) were used to construct the HDP1 (Supplemental Table 6). HDP3, composed of fruits and dairy products, was also not significantly associated with T2D risk (pooled IRR per SD = 0.976, 95% CI 0.948–1.005, Table 3), when using the secondary outcome definition. For the remaining HDPs (2, 4–6) the pooled risk estimators did not indicate associations with T2D risk (Table 3). Overall, there was moderate to substantial heterogeneity (*I*^2^ = 58–83%, Table 3) for the HDP–T2D associations. For HDP1, none of the characteristics (age, BMI, follow-up time and region) explained the observed heterogeneity (*I*^2^ = 66%) in meta-regressions (data not shown).

**Table 3:** Pooled findings of federated random effect meta-analyses to test for the association between the simplified healthy and unhealthy dietary pattern variables (per one standard deviation) (cut-off factor loadings > 0.2) and incident type 2 diabetes across InterConnect cohorts

DP variables	Outcome definition	IRR^a^ [95% CI]	*I*^2^	Tau^2^	*p* value
HDP1	Primary	**1.057 [1.027–1.088]**	66%	0.002	< 0.01
	Secondary	**1.042 [1.018–1.065]**	58%	0.002	< 0.01
HDP2	Primary	1.003 [0.970–1.038]	83%	0.004	< 0.01
	Secondary	0.999 [0.974–1.026]	77%	0.003	< 0.01
HDP3	Primary	0.995 [0.963–1.027]	81%	0.004	< 0.01
	Secondary	0.976 [0.948–1.005]	79%	0.004	< 0.01
HDP4	Primary	1.030 [0.993–1.067]	83%	0.005	< 0.01
	Secondary	1.023 [0.994–1.052]	79%	0.003	< 0.01
HDP5	Primary	1.023 [0.992–1.054]	77%	0.003	< 0.01
	Secondary	1.015 [0.990–1.040]	72%	0.002	< 0.01
HDP6	Primary	1.030 [0.995–1.065]	79%	0.004	< 0.01
	Secondary	1.020 [0.994–1.047]	72%	0.003	< 0.01
UDP1	Primary	1.002 [0.973–1.033]	49%	0.002	< 0.01
	Secondary	0.991 [0.964–1.018]	55%	0.002	< 0.01
UDP2	Primary	1.032 [0.994–1.073]	76%	0.005	< 0.01
	Secondary	1.027 [0.996–1.059]	73%	0.004	< 0.01
UDP3	Primary	**1.104 [1.059–1.151]**	85%	0.006	< 0.01
	Secondary	**1.094 [1.056–1.133]**	84%	0.006	< 0.01
UDP4	Pimary	**1.070 [1.044–1.098]**	49%	0.001	< 0.01
	Secondary	**1.055 [1.031–1.079]**	55%	0.002	< 0.01
UDP5	Primary	**1.044 [1.014–1.075]**	65%	0.002	< 0.01
	Secondary	**1.033 [1.006–1.061]**	67%	0.002	< 0.01
UDP6	Primary	**1.045 [1.013–1.078]**	81%	0.003	< 0.01
	Secondary	**1.039 [1.014–1.065]**	76%	0.002	< 0.01
UDP7	Primary	**1.044 [1.009–1.080]**	71%	0.003	< 0.01
	Secondary	1.031 [0.999–1.064]	75%	0.004	< 0.01

*CI* confidence intervals, *FL* factor loading, *HDP* healthy dietary pattern, *IRR* incidence rate ratios, *I*^2^ inconsistency value, *n.a.* not applicable, *UDP* unhealthy dietary pattern

^a^Association is adjusted for age, sex, BMI, physical activity, education, smoking, alcohol consumption, total energy intake and hypertension; significant IRRs (95% CI not comprising IRR=1.000) are highlighted in bold

### Unhealthy dietary patterns and risk of T2D

Five of the seven UDPs (UDP3-7) were associated with a higher T2D risk in pooled analyses across all cohorts (Table 3, Figs. 1, 2, Supplemental Table 6, Supplemental Figs. 2–5). The UDP 3–7 included mostly meat products, French fries and refined grains (Table 2). Only UDP 6 differed from these DPs, as meat products were not included, but soft drinks and the components whole grains, vegetables, fruits and legumes (including soy) with negative weightings were included. UDP 3 showed the strongest association with incident T2D (primary outcome: pooled IRR per 1 SD = 1.104, 95% CI 1.059–1.151; secondary outcome: pooled IRR per 1 SD = 1.094, 95% CI 1.056–1.133 for UDP3 based on FL ≥ 0.2). However, heterogeneity was substantial across studies (*I*^2^ = 85% and 84%). The region partly explained heterogeneity for UDP3 (16%) in meta-regression. When UDP3 was constructed using FGs with FL ≥ 0.4, only red meat remained as component and associations were considerably weaker, although still statistically significant (Supplemental Table 6). Most cohort-specific IRRs indicated that UDP3 was associated with a higher T2D risk or a trend towards an association (Figs. 1, 2). Similar findings, although weaker, were observed for UDPs 4–7, where heterogeneity ranged from moderate (*I*^2^ = 49% for UDP 4) to substantial (*I*^2^ = 81% for UDP 6). Here, region explained a considerable proportion of the heterogeneity for UDP6 (29%) and UDP7 (25%), while follow-up time explained 30% for UDP5 and 24% for UDP6 of the overall heterogeneity. No association with T2D risk was found for UDP 1 and UDP 2, neither for the two outcome definitions nor for the two FL cut-offs (Table 3, Supplemental Table 6).

### Dietary patterns with “mainly healthy” and “mainly unhealthy” food groups and T2D risk

**Fig. 3:**
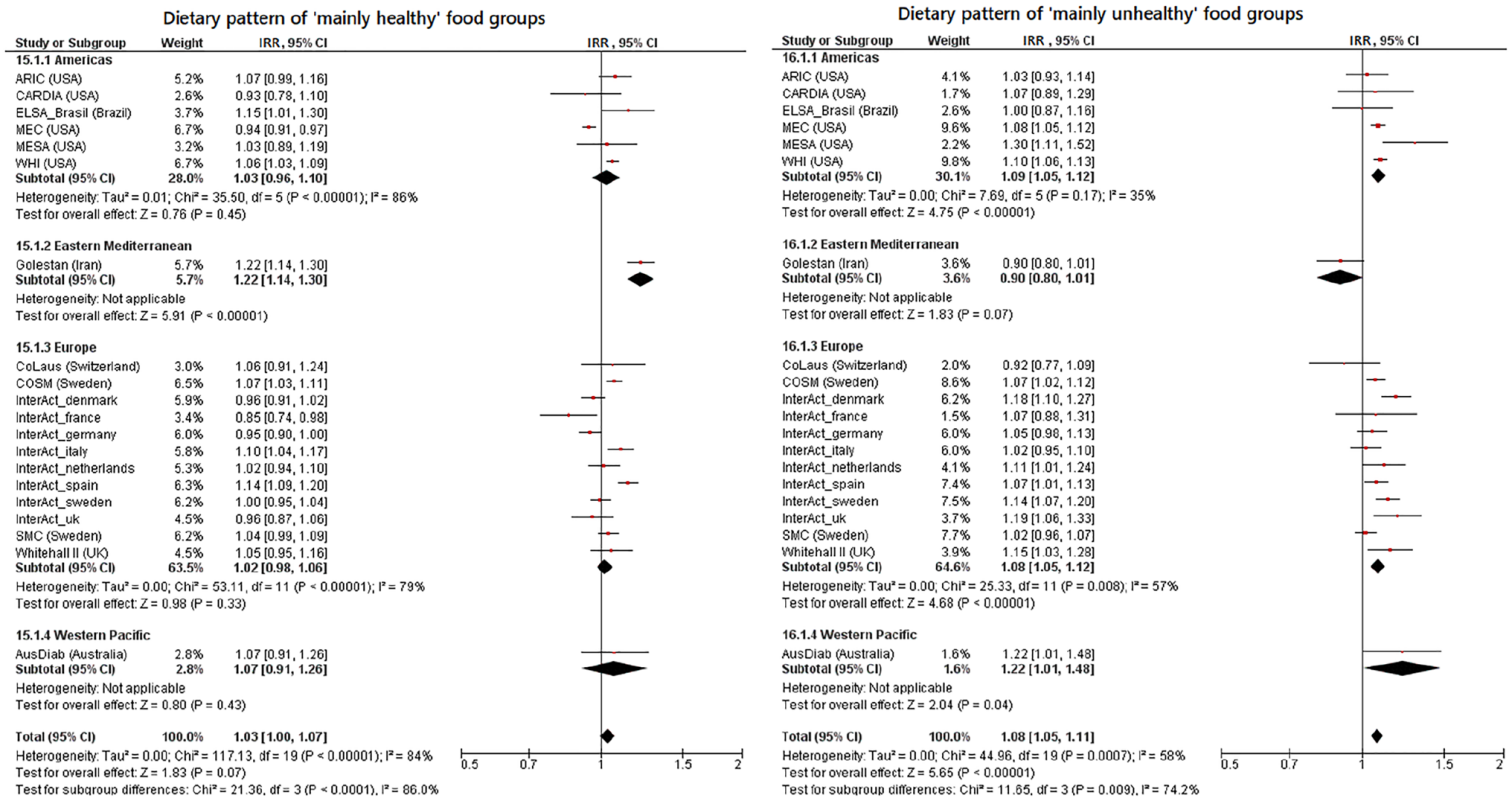
Incidence rate ratios and 95% confidence intervals for the association between the dietary patterns of “mainly healthy” and “mainly unhealthy” food groups and incident type 2 diabetes using the primary outcome. Associations are shown by subgroups of region and adjusted for age, sex, BMI, physical activity, education, smoking, alcohol consumption, total energy intake and hypertension. *CI* confidence intervals, *IRR* incidence rate ratios

We evaluated the two DPs reflecting previously published DPs with overlapping FG components irrespective of whether they have been described to be associated with T2D previously or not [1]. The DP consisting of “mainly healthy” FGs, i.e. fruits, vegetables, legumes, poultry and fish, was not associated with T2D risk across the included cohorts (primary outcome: pooled IRR per 1 SD = 1.033, 95% CI 0.998–1.071; secondary outcome: pooled IRR per 1 SD = 1.000, 95% CI 0.975–1.026) (Fig. 3, Supplemental Fig. 6). The heterogeneity across studies was substantial (primary outcome: *I*^2^ = 84%, secondary outcome: *I*^2^ = 76%). Hence, the forest plots show the cohorts arranged by region. In contrast, the DP consisting mainly of “mainly unhealthy” FGs, i.e. refined grains, French fries, red meat, processed meat, high-fat dairy products and eggs, was significantly associated with a higher T2D risk (primary outcome: pooled IRR per 1 SD = 1.079, 95% CI 1.051–1.108; secondary outcome: pooled IRR per 1 SD = 1.067, 95% CI 1.037–1.098) (Fig. 3, Supplemental Fig. 6). The heterogeneity was moderate for the primary outcome (*I*^2^ = 58%), but substantial for the secondary outcome (*I*^2^ = 74%). Most study-specific IRRs indicated a higher risk of this DP, except for the Golestan Cohort Study, which pointed towards an inverse association.

### Sensitivity analysis of UDP 3

UDP3 was composed of the FGs red meat, processed meat, poultry, eggs, fish, French fries, refined grain products, and rice. To assess the contribution of these individual FGs to the T2D risk of UDP3, a sensitivity analysis was carried out by excluding individual FGs (Supplemental Table 7). The exclusion of refined grains resulted in the highest reduction of the IRR estimate (from 1.094–1.047, − 4.74%), followed by processed meat (− 1.66%) and eggs (− 1.10%).

## Discussion

This study investigated associations between exploratory DPs and T2D risk in a large number of prospective cohort studies in a worldwide context, using harmonized data analyses across all studies and federated meta-analyses of individual studies. No robust inverse associations were observed between HDPs and risk of T2D. HDP1 was associated with a higher T2D risk in primary analysis, but this unexpected finding was not confirmed in sensitivity analyses. We observed more consistent findings for UDPs with five of the seven UDPs being associated with higher T2D risk in our meta-analysis of included studies. We investigated two DPs which reflect commonly shared FGs of exploratory DPs identified in previous studies on DP and T2D. The DP with “mainly healthy” FGs, characterized by higher intakes of vegetables, legumes, fruits, poultry and fish, was not associated with T2D risk, but the DP with “mainly unhealthy” FGs, characterized by red meat, processed meat, high-fat dairy products, eggs, refined grains and French fries, was associated with a higher T2D risk. The effect size for all the significant associations was relatively modest with IRRs being 1.10 per 1 SD increased DP score or less.

Previous studies have shown differences in risk associations between DPs and T2D in U.S. cohorts and the European EPIC-InterAct study, although this was restricted to a priori DPs like the Dietary Approaches to Stop Hypertension (DASH) diet, the Alternative Healthy Eating Index (AHEI) or reduced rank regression-derived DPs [1, 43]. Given the strong heterogeneity in the composition of exploratory DPs already in the European context, this underlines the importance of investigating if population-specific DP–T2D associations can be replicated across diverse populations, where even higher heterogeneity is expected. To our knowledge, this is the first study to investigate if associations of exploratory DPs with T2D risk can be replicated across cohorts from multiple regions across the world.

We have previously investigated the generalizability of exploratory DPs associations with T2D in EPIC-InterAct, a European-wide cohort study [13]. In this analysis, three DPs identified in country-specific analyses were associated with T2D. However, only one DP was consistently associated with T2D risk across the included European cohorts (pooled IRR per 1 SD: 1.12, 95% CI 1.04–1.20). This DP was characterized by high intakes of processed meat, potatoes (including French fries), vegetable oils, sugar, cake and cookies, and tea. Besides the EPIC-InterAct study, we are not aware of any further systematic replication of associations of exploratory DPs and T2D. Also, the EPIC-InterAct study did not attempt to replicate T2D-associated DPs identified in other cohorts than EPIC-InterAct, which has been our current major aim.

We were able to replicate associations with higher T2D risk for five of seven investigated UDPs. These five UDPs (UDP3-7) share red meat, processed meat, French fries and refined grains (comprising refined grain bread and refined grain breakfast cereals) as component FGs. Also eggs and high-fat dairy products were component FGs of three out of these five DPs. These FGs are identical to those which we used to construct one DP based on commonly shared “mainly unhealthy” FGs of published DPs [1]. Consequently, this pattern was also associated with a higher T2D risk in our meta-analysis: we observed a pooled IRR of 1.08 per 1 SD, 95% CI 1.05–1.11 for the primary outcome definition, being slightly stronger than the risk estimates for most of the UDPs, which ranged between pooled IRRs of 1.04 for the UDP5 by Yu et al. [7] and for UDP7 by Schoenaker et al. [9] to 1.07 for the UDP4 identified by Erber et al. [6]. However, an even higher risk estimate was found for UDP3 (IRR of 1.10 per 1 SD, 95% CI 1.06–1.15), which had been observed in the Melbourne Collaborative Cohort Study to be associated with higher risk of T2D [5]. This DP was not only characterized by red and processed meat, eggs, French fries, refined grains, but also by fish, poultry and rice. We noted that the DPs associated with higher risk in our meta-analyses had only potatoes (including French fries) and processed meat in common with the DP identified to be associated in the EPIC-InterAct study [13]. To gain insight into the role of individual FGs for pattern associations, we conducted a sensitivity analysis on the UDP3-T2D association by excluding individual FGs one at a time. Particularly the exclusion of refined grains led to an attenuation of the risk estimate from IRR of 1.10 to 1.05 for the primary outcome. Still, other components seemed to contribute to the associations and we interpret the synergy of these component FGs in this pattern as driving the association with T2D. The UDPs which were identified as being associated with a higher risk of T2D did not only show overlaps but also differences in component FGs. For example, butter (UDP4), sugar and confectionary and offals (UDP5) or pizza (UDP6, UDP7) were pattern-specific components besides the commonly shared FGs. Two of the UDPs (UDP5, UDP6) additionally shared the FG sugar-sweetened beverages. This food group was also a component in 4 out of 5 previously identified reduced rank regression-patterns, which were associated with higher T2D risk [14, 44–46] and evidence from a systematic literature review suggests 13% risk increase for T2D per one serving (250 mL/day), even after adjustment for BMI [47]. The UDP6 was furthermore characterized by the negatively weighted FGs cakes & cookies, legumes, vegetables, fruits and whole grains. However, after exclusion of these FGs due to the use of the cut-off FL ≥ 0.4, the IRR was only marginally changed.

None of the HDPs, either individual DPs described by single studies or the DP defined by commonly shared “mainly healthy” FGs of investigated patterns, were inversely associated with T2D risk in our meta-analyses. This is generally in line with evidence for single FGs being components of such DPs. For instance, vegetables, fruits, legumes, poultry and fish have not been clearly identified to relate to lower T2D risk in cohort studies [48]. In contrast to the original observation from the Finnish Mobile Clinic Health Examination Survey [4], we observed the HDP1 being associated with a higher risk of T2D. Red meat and eggs—frequent components of UDPs—were also contributing components of this pattern; thus, the direction of association in our analysis could potentially be driven by these two components. While a higher T2D risk of red meat is well documented [48], the role of egg consumption remains unclear [49]. Differences how specific foods are prepared and/or consumed together across populations may explain their association with healthy or unhealthy patterns. Furthermore, if a food group like fish is the main animal protein source in a population, detrimental components like methylmercury could play a more important role leading to health detrimental effects than in a population, where these components play a minor role due to less intake [50].

Besides the components of the investigated DPs, it is relevant to discuss overall methodological limitations. To enable the meta-analytical investigation of the DPs across so many different cohorts in the first place, we harmonized the cohort specific food items into a number of food groups. This inherits the problem of summarizing different numbers of food items into one food group, depending on the original dietary assessment. Hence, the difference in median intake of certain food groups between the cohorts could be due to real dietary intake differences in the populations or due to a higher extent of inquired food items. Furthermore, the condensing of food items into food groups led to a lack of granularity. Hence, potential differences in the association with T2D of specific food items, e.g. green leafy vegetables [51], could not be distinguished from other food items within this food group. Another methodological limitation could be the lack of detail about preparation methods, e.g. frying, in the dietary assessment of most of the participating cohorts. Hence, this may have led to an underestimation of the association for the UDP3, which related to each of fried fish, poultry and rice in the original study by Hodge et al. [5], while we could only consider overall intake of fish, poultry and rice in our study. A distinction between French fries and potatoes (non-fried) was also not possible in all participating cohorts. However, a recent meta-analysis investigated the association of potatoes with T2D risk and distinguished between French fries and boiled/baked/mashed potatoes and both types of potato culinary preparations were associated with a higher T2D risk, although to a higher extent for 150 g/day intake of French fries (RR of 1.66, 95% CI 1.43–1.94) compared to 150 g/day intake of boiled potatoes (RR of 1.09, 95% CI 1.01–1.18) [52]. Hence, we would still expect the risk estimates to point to a similar direction. Besides the food items, a common set of important and well-established confounders had to be harmonized across the cohorts. The set was selected based on those confounders, which were reported in the original publications of DPs and based on the availability of confounders in the participating InterConnect cohorts. Clearly, due to the harmonization approach and the technical setup for federated data analysis, it was not possible to account for all potential confounders, either being generally important (e.g. family history of diabetes) or being relevant for some specific study populations (e.g. ethnicity). Still, the consideration of a harmonized confounder set could be seen as strength of this study. Alongside the exposure and covariates, the outcome definitions needed also harmonization attempts. Due to different definitions of T2D as outcome in the participating cohorts, we have applied two different outcome definitions (primary, secondary). To assess if large differences in the number of T2D cases in some cohorts due to the definitions affect the associations, we conducted a sensitivity analysis. We compared the IRR for subgroup analyses of cohorts with a large (> 40%) to small (≤ 40%) difference and did observe slightly attenuated associations for all UDPs (data not shown). This indicated that a stricter outcome definition (“primary outcome”) resulted in slightly stronger associations.

Furthermore, the DPs were replicated in the different cohorts by using a simplification process which restricts the DP score calculations to those FGs with high FL and ignores differences in FL between FGs [17]. However, many original DPs contained only very few FGs with relative high FL (≥ 0.4). So, for instance, the simplified UDP3 resulted in red meat as the only FG and hence lost the complex pattern structure. Therefore, we decided to use FGs in the simplified pattern with FL ≥ 0.2 as the main analysis. The simplification ignores relative differences in contributions of FGs to DPs (reflected by differences in FLs), however, it supports interpretation of DPs in terms of FG intake [17]. While the approach has been successfully applied to replicate other data-driven pattern associations [14, 43], we cannot rule out that the relative loss in precision in DP score calculation has influenced the success of pattern-T2D association replications in our study.

We observed moderate to strong heterogeneity of associations across cohorts, with I^2^ values ranging from 49% (UDP4) to 85% (UDP3). Heterogeneity between studies may have different explanations. The condensation of foods into harmonized FGs in the cohorts may have led to the inclusion of heterogeneous food items due to strong culinary differences between populations, but also due to different extent of inquired food items depending on the dietary assessment instrument. Another explanation for heterogeneity could have been the inclusion of cohorts with a short follow-up time, introducing the bias of reverse causation. Especially for HDPs, participants with a high risk at developing T2D could have changed their dietary habits by eating more health promoting food groups, but still developed the disease. However, this could not be confirmed by the results of our meta-regression on several characteristics of the cohorts (region, follow-up time, age, BMI). Here, the follow-up time explained only a considerable proportion of heterogeneity for two UDPs (UDP5, UDP6). Overall, the magnitude of the pooled risk estimates was much smaller compared to the original studies. However, comparability is constrained, since the risk estimates are given per 1 SD increase and SD is highly dependent on the population distribution of the respective DPs. Nevertheless, we were restricted to the calculation of analyses assuming a linear association between the DPs and T2D, due to the federated approach and the solutions, which could be realised with DataSHIELD. Hence, generalizable conclusions based solely on the magnitude of risk estimates from the meta-analyses should be done with caution and no quantitative recommendations can be deduced for public health guidance. Therefore, we mainly base our conclusions on the consistency of direction of associations: in the meta-analyses with significant pooled risk estimates, the majority of included cohorts pointed also towards a higher risk. Another limitation was the standardization of FGs for DP score calculation based on the distribution of FG intake in the respective cohorts. This could be a problem, if food intake distributions differ extensively between those cohorts compared to the study population where a DP had previously been reported from and hence, may jeopardize attempts to replicate associations of DPs with disease risk. However, two main reasons were pivotal for this approach. On the one hand, the information on the intake distribution was not provided in most original publications, but rather the correlation structure as a basis for the exploratory derivation of DPs. On the other hand, even if this information would be provided by the original publications, this would result in more limitations: In most studies, non- or semi-quantitative dietary assessment instrument were applied and hence, the reported intake distributions did not provide a valid estimation of absolute intakes. Furthermore, dietary assessment instruments per se differed between the cohorts and nothing is known about their comparability in estimating food intake. Another limitation of this study was the high exclusion rate of 46.9%. Hence, a potential selection bias due to missing follow-up time, covariates or food intake data could not be ruled out.

## Conclusion

To our knowledge, this is the first study replicating population-specific associations of exploratory DPs with T2D risk across a large number of cohort studies from different continents. Our meta-analyses of harmonized individual-level data from various cohorts revealed a higher T2D risk for several DPs characterized by higher intake of red meat, processed meat, French fries and refined grains (comprising refined grain bread and refined grain breakfast cereals). These results confirm former study-specific results in a generalizable context and therefore enrich evidence for DPs related to higher T2D risk. However, none of the inverse associations of investigated HDPs could be confirmed across different cohorts.

## Supplementary Information

Below is the link to the electronic supplementary material.

Supplementary file1 (DOCX 7877 KB)

Supplementary file2 (XLSX 19 KB)

## Data Availability

Due to the federated and collaborated design of this InterConnect study, data and material cannot be made accessible. Individual study meta-data may be available upon request from the individual study PI’s.

## Code availability

The analysis code can be provided on request.
