# Supplementary material for: Associations between exploratory dietary patterns and incident type 2 diabetes: a federated meta-analysis of individual participant data from 25 cohort studies
Source: Eur J Nutr. 2022 Jun 1;61(7):3649–67. doi: 10.1007/s00394-022-02909-9 (PMC9464116; doi:10.1007/s00394-022-02909-9)
Supplement: Supplementary file 1 — Supplementary file1 (DOCX 7877 KB) [file 394_2022_2909_MOESM1_ESM.docx]

**Supplementary Data**

**Study names of participating InterConnect cohorts:**

ARIC - Atherosclerosis Risk in Communities study

AusDiab - The Australian Diabetes, Obesity and Lifestyle Study

# CARDIA - Coronary Artery Risk Development in Young Adults Study

CoLaus - The Cohorte Lausannoise

COSM - The Cohort of Swedish Men

ELSA-Brasil - The Brazilian Longitudinal Study of Adult Health

EPIC-InterAct - European Prospective Investigation into Cancer - InterAct study

Golestan - The Golestan Cohort Study

KoGES_CAVAS - Korean Genome and Epidemiology Study_Cardiovascular Disease Association

KoGES_ASAS - Korean Genome and Epidemiology Study_Ansan and Ansung

MEC - The Multi Ethnic Cohort

MESA - The Multi-Ethnic Study of Atherosclerosis

MTC - The Mexican Teachers Cohort

PRHHP - The Puerto Rico Heart Health Program

SUN - The University of Navarra Follow-up Study

SMC – The Swedish Mammography Cohort

WHI-OS - The Women’s Health Initiative

Whitehall II– The Whitehall II Study

**Supplemental Table 1:** Characteristics and assessment of diet of InterConnect cohorts

| Study name  (Country) | Original inclusion/exclusion criteria | Recruitment  time-frame | Baseline sample | Dietary assessment | Units provided |
| --- | --- | --- | --- | --- | --- |
| ARIC  (USA) | Ethnically-representative 45-64 years in 4 communities | 1987 - 1989 | 15,792 | Interviewer-administered semi-quantitative food frequency questionnaire | portion size and frequency |
| AusDiab (Australia) | ≥25 years and resident at the address for 6 months | 1999 - 2000 | 11,247 | Semi-quantitative food frequency questionnaire | Grams per day |
| CARDIA (USA) | Black and white men and women aged 18-30 years selected to be representative of four cities | 1985 - 1986 | 5,100 | Interviewer-administered dietary history | portion size per day |
| CoLaus  (Switzerland) | Men and women aged 35-75 years living in Lausanne | 2009 - 2012 | 5,064 (1. follow-up) | Validated self-administered semi quantitative food frequency questionnaire of 97 items covering last 4 weeks before examination. | Grams per day |
| COSM (Sweden) | Men, aged 45-79 years, living in Västmanland and Örebro counties | 1997 - 1998 | 45,906 | self-administered food frequency questionnaire | Grams per day |
| ELSA-Brasil  (Brasil) | active or retired employees of 6 institutions aged 35–74 years | 2008 - 2010 | 15,105 | Interviewer-administered online food frequency questionnaire | Grams per day |
| EPIC-InterAct  (Europe) | Case-cohort of all T2D cases occurring in EPIC cohorts between 1991 and 2007 and a subcohort of individuals randomly selected from those with available stored blood and buffy coat, stratified by centre. | 1991 - 1998 | 12,403 (cases); 16,154 (subcohort) | Quantitative dietary questionnaire with individual average portion sizes (in France, Spain, Netherlands, Germany, and Italy, except Naples) or a semi-quantitative FFQ (in Denmark, Naples (Italy), Sweden, and the UK), developed and validated locally. | Grams per day |
| Golestan  (Iran) | Healthy individuals aged 40-75 years, 80% from rural areas and 75% of Turkmen ethnicity living in Golestan | 2004 - 2008 | 50,000 | Semi-quantitative food frequency questionnaire | Grams per day |
| KOGES_ASAS (South Korea) | Men and women aged ≥ 40 years in national health examinee registry | 2001-2002 | 10,030 | Semi-quantitative food frequency questionnaire | Grams per day |
| KOGES_CAVAS (South Korea) | Men and women aged ≥ 40 years in national health examinee registry | 2005-2011 | 21,715 | Semi-quantitative food frequency questionnaire | Grams per day |
| MESA  (USA) | Ethnically-stratified asymptomatic men and women aged 45-84 years | 2000 - 2002 | 6,814 | Self-administered, modified-Block food frequency questionnaire | portion size per day |
| MEC  (USA) | Multi-ethnical individuals aged 45-75 years living in Hawaii and Los Angeles | 1993 - 1996 | 215,831 | self-administered quantitative food frequency questionnaire | Grams per day |
| MTC (Mexico) | female public school teachers residing in a culturally, geographically and economically diverse 12-state area | 2006 - 2008 | 115,314 | Semi-quantitative food frequency questionnaire | portion size and frequency |
| PRHHP (USA) | Men aged 45-64 years representing the urban/rural composition of the island (70% urban and 30% rural) | 1965 | 8,793 | 24-hour recall | portion size per day |
| SUN (Spain) | University graduates from the University of Navarra and professional associations, willing to commit themselves for returning questionnaires every 2 years. | 2000 - ongoing | 4,717 (2000); 16,390 (2005) | Semi quantitative food frequency questionnaire | Grams per day |
| SMC (Sweden) | Women living in Uppsala county born 1914-1948 and Västmanland County born 1917-1948 | 1987-1990 | 61,433 | Self-administered food frequency questionnaire | Grams per day |
| WHI-OS (USA) | postmenopausal women aged 50–79 years | 1993 and 1998 | 93,676 | Standardised self-administered food frequency questionnaire | portion size per day |
| Whitehall II (UK) | civil servants aged 35–55 years working in the London offices of 20 Whitehall departments | 1985-1988 | 10,308 | Food frequency questionnaire | portion size per day |

**Supplemental Table 2:** Composition of published risk reducing T2D-associated dietary patterns and replication in InterConnect

| Author, year, study name | published risk estimator (95% confidence interval) | Food items (factor loadings) of published pattern | InterConnect food groups used to replicate pattern | InterConnect DP variable |
| --- | --- | --- | --- | --- |
| Montonen, 2004  *Finnish Mobile Clinic Health Examination Survey* | RR:  0.72 (0.53 - 0.97)  p = 0.03 | **Yellow and red vegetables (0.64)**  **Green vegetables (0.63)**  **Vegetables, other (0.57)**  **Fruit (0.62)**  **Berries (0.28)**  Poultry (0.35)  Eggs (0.34)  Red meat (0.32)  Whole milk (- 0.30)  Peas and nuts (0.23)  Regular dairy products (0.26)  Reduced-fat dairy (0.26)  Margarine and oil (0.26)  canned/frozen Fish (0.22) | Vegetables  Fruits  Poultry  Eggs  Red meat  Whole milk  Nuts  High fat dairy  low-medium fat dairy  Margarine  Fish | HDP 1 |
| Erber, 2010  *Multiethnic cohort* | HR men:  0.86 (0.77 - 0.95)  p = 0.004  HR women:  1.02 (0.91 - 1.14)  p = 0.93 | **Dark-green vegetables (0.87)**  **Deep-yellow vegetables (0.79)**  **Other vegetables (0.86)**  **Citrus fruits, melons, and berries (0.36)**  **Other fruits (0.44)** | Vegetables  Fruits | HDP 2 |
| Erber, 2010  *Multiethnic cohort* | HR men:  0.92 (0.83 - 1.02)  p = 0.04  HR women:  0.85 (0.76 - 0.96)  p = 0.005 | **Milk and yogurt (0.71)**  **Citrus fruits, melons, and berries (0.71)**  **Other fruits (0.71)**  Cheese (0.35) | Whole Milk, High-fat dairy, medium/low-fat dairy  Fruits  Cheese, low-fat cheese | HDP 3 |
| Odegaard, 2011  *Singapore Chinese Health Study* | HR never smoker:  0.77 (0.65 - 0.92)  p = 0.001  HR smoker:  1.17 (0.91 - 1.51)  p = 0.39 | **Vegetables (0.20-0.54)**  **Potatoes without fries (0.41)**  **Legumes/ soy food (0.20-0.46)**  Fruits (0.27-0.34)  Fish (0.22-0.27)  Poultry (0.25) | Vegetables  Potatoes  Legumes/ soy  Fruits  Fish  Poultry | HDP 4 |
| Yu, 2011  *Hong Kong Dietary Survey* | OR  0.76 (0.58 - 0.99)  p = NA | **Fish and seafood (0.72)**  **Fruit (0.66)**  **Other vegetables (0.60)**  **Dark green and leafy vegetables (0.47)**  Cruciferous vegetables (0.27)  Tomatoes (0.20)  Legumes (0.32)  Soya (0.28)  Mushrooms and fungi (0.30)  Nuts (0.22) | Fish, Shellfish  Fruits  Vegetables  Legumes/Soy  NA (Mushrooms and fungi)  Nuts | HDP 5 |
| Morimoto, 2012  *rural Japanese population study* | HR:  0.78 (0.61-0.95)  p = 0.008 | **Green vegetables (0.62)**  **Other vegetables (0.55)**  **Potatoes (0.56)**  **Seaweeds (0.53)**  **Fruits (0.48)**  Soybean products (0.38)  Dressing oil and butter (0.29)  Miso soup (0.27)  Pickles (0.22)  Fish (0.20) | Vegetables  Potatoes without fries  NA (Seaweeds)  Fruits  Legumes/Soy  Butter, margarine, mayonnaise  NA (Miso soup)  NA (Pickles)  Fish, Shellfish | HDP 6 |

DP – dietary patterns; HDP – healthy dietary pattern; HR – hazard ratio; NA – not available; OR – odds ratio; RR – relative risk

**Supplemental Table 3:** Composition of published risk enhancing T2D-associated dietary patterns and replication in InterConnect

| Author, year, study name | published risk estimator (95% confidence interval) | published food groups (factor loadings) of the respective pattern | InterConnect food groups used to replicate pattern | InterConnect DP variable |
| --- | --- | --- | --- | --- |
| van Dam, 2002  *Health Professionals Follow-up Study* | RR men:  1.59 (1.32 - 1.93)  p < 0.001 | **Red meat (0.64)**  **Processed meat (0.61)**  **Refined grains (0.48)**  **French fries (0.48)**  **High-fat dairy products (0.47)**  **Sweets and desserts (0.42)**  **Eggs (0.42)**  **Condiments (0.41)**  High-sugar drinks (0.36)  Snacks (0.36)  Butter (0.34)  Mayonnaise (0.34)  Potatoes (0.29)  Margarine (0.29)  Pizza (0.29)  Coffee (0.27)  Chowder or cream soup (0.22)  Nuts (0.22) | Red meat  Processed meat  Refined grains, pasta, rice  French fries  Whole milk, high fat-diary, cheese, ice cream  Cake, Confect  Eggs  Sugar & Confectionary, condiment sauces  Sugary soft drinks  NA (Snacks)  Butter  Mayonnaise  Potatoes  Margarine  Pizza  Coffee  NA (Chowder or cream soup)  Nuts | UDP 1 |
| Montonen, 2004  *Finnish Mobile Clinic Health Examination Survey* | RR:  1.49 (1.11 - 2.00)  p = 0.01 | **Butter (0.68)**  **Potatoes (0.66)**  **Whole milk (0.59)**  **Red meat (0.56)**  **Jams and sugar-rich condiments (0.49)**  **Rye (0.45)**  **Grain other than rye or wheat (0.43)**  **Processed meat (0.43)**  Wheat (0.39)  Eggs (0.32)  Fish, salted or smoked (0.31)  Fish, unprocessed (0.31)  Peas and nuts (0.30) | Butter  Potatoes, French fries  Whole milk  Red meat, Offals  Sugar & Confectionary  Whole grain products  Rice, whole grain cereals  Processed meat  Pasta, refined grains  Eggs  Fish  Shellfish  Nuts, Legumes, Soy | UDP 2 |
| Hodge, 2007  *Melbourne Collaborative Cohort Study* | OR  1.65 (1.03 - 2.63)  p = 0.24 | **Red meat (0.27-0.41)**  Potato cooked in fat (0.36)  Savory pastries (0.34)  Fried egg and Egg dish (0.32)  Fried fish (0.31)  Processed meat (0.20-0.31)  White bread (0.29)  Roast/fried chicken (0.28)  Fried rice (0.25) | Red meat  French fries  NA (Savory pastries)  Eggs  Fish  Processed meat  Refined grain products  Poultry  Rice | UDP 3 |
| Erber, 2010  *Multiethnic cohort* | HR men  1.40 (1.23–1.60)  p = <0.0001  HR women  1.22 (1.06–1.40)  p = 0.004 | **Discretionary fat (0.88)**  **Meat and organ meat (0.83)**  **Frankfurters, sausage, luncheon meat (0.72)**  **White potatoes (0.68)**  **Non-whole grains (0.67)**  **Eggs (0.63)**  **Cheese (0.63)** | Butter, margarine  Red meat, offals  Processed meat  Potatoes, French fries  Refined grains  Eggs  Cheese, low-fat cheese | UDP 4 |
| Yu, 2011  *Hong Kong Dietary Survey* | OR  1.39 (1.04 – 1.84)  p = NA | **Red meats (0.92)**  **Milk (0.44)**  Condiments (0.38)  Eggs (0.37)  Refined grains (0.37)  Beverages (0.37)  Poultry (0.35)  Organ meat (0.27) | Red meat, processed meat  High fat-diary, medium/low-fat diary, cheese, low-fat cheese, ice  Sugar & Confectionary  Eggs  Refined grains, pasta, white rice  Sugary soft drinks  Poultry  Offals | UDP 5 |
| Bauer, 2012  *EPIC-Netherlands* | HR Q4 vs Q1  1.70 (1.31 - 2.20)  p ≤ 0.0001 | **Soft drinks with sugar (0.76)**  **Other non-alcoholic drinks (0.72)**  **Soft drinks sugar free (0.63)**  **French fries (0.62)**  **Snacks (0.51)**  **Fruit (- 0.47)**  Low-fat dairy products (- 0.36)  Cereals with high amount of fiber (- 0.34)  Bread with low amount of fiber (0.34)  Boiled vegetables and legumes (- 0.33)  Cakes and cookies (- 0.28)  Raw vegetables (- 0.28) | Sugary soft drinks  NA (Other non-alcoholic drinks)  Low sugary soft drinks  French fries  Pizza  Fruits  medium/low-fat diary  Whole grain products and whole grain cereals  refined grain bread  Legumes  Cakes  Vegetables | UDP 6 |
| Schoenaker, 2013  *Australian Longitudinal Study on Womens Health* | OR women  1.73 (1.12 - 2.67)  p=0.003 | High fat dairy (0.35)  White bread (0.28)  Red meat (0.27)  Processed meat (0.26)  Take-away food (0.24)  Potatoes with fat (0.21)  Snacks factor (0.20) | Whole milk, cheese  Refined grain bread  Red meat  Processed meat, Hamburger  Pizza  French fries  Cakes, ice cream, Confectionary | UDP 7 |

DP – dietary pattern; HR – hazard ratio; NA – not available; OR – odds ratio; RR – relative risk; UDP – unhealthy dietary pattern

**Supplemental Table 4:** Overview of potential confounders used in the original publications and availability across InterConnect cohorts

|  | potential Confounder | HDP 1 | HDP 2 | HDP 3 | HDP 4 | HDP 5 | HDP 6 | UDP1 | UDP2 | UDP3 | UDP4 | UDP5 | UDP6 | UDP7 | ARIC | AusDiab | CARDIA | CoLaus | COSM/SMC | ELSA-Brasil | EPIC-InterAct | Golestan | KOGES | MEC | MESA | MTC | PRHHP | SUN | WHI_OS | Whitehall2 |
| --- | --- | --- | --- | --- | --- | --- | --- | --- | --- | --- | --- | --- | --- | --- | --- | --- | --- | --- | --- | --- | --- | --- | --- | --- | --- | --- | --- | --- | --- | --- |
| used confounder | Age | ✓ | ✓ | ✓ | ✓ | ✓ | ✓ | ✓ | ✓ | ✓ | ✓ | ✓ | ✓ | 🗶 | ✓ | ✓ | ✓ | ✓ | ✓ | ✓ | ✓ | ✓ | ✓ | ✓ | ✓ | ✓ | ✓ | ✓ | ✓ | ✓ |
|  | Sex | ✓ | 🗶 | 🗶 | ✓ | ✓ | ✓ | 🗶 | ✓ | ✓ | 🗶 | ✓ | ✓ | 🗶 | ✓ | ✓ | ✓ | ✓ | ✓ | ✓ | ✓ | ✓ | ✓ | ✓ | ✓ | W | ✓ | ✓ | W | ✓ |
|  | BMI | ✓ | ✓ | ✓ | ✓ | ✓ | ✓ | ✓ | ✓ | ✓ | ✓ | ✓ | ✓ | ✓ | ✓ | ✓ | ✓ | ✓ | ✓ | ✓ | ✓ | ✓ | ✓ | ✓ | ✓ | ✓ | ✓ | ✓ | ✓ | ✓ |
|  | Physical activity | 🗶 | ✓ | ✓ | ✓ | ✓ | ✓ | ✓ | 🗶 | 🗶 | ✓ | ✓ | ✓ | ✓ | ✓ | ✓ | ✓ | ✓ | ✓ | ✓ | ✓ | ✓ | ✓ | ✓ | ✓ | ✓ | ✓ | ✓ | ✓ | ✓ |
|  | education | 🗶 | ✓ | ✓ | ✓ | 🗶 | 🗶 | 🗶 | 🗶 | 🗶 | ✓ | 🗶 | ✓ | ✓ | ✓ | ✓ | ✓ | ✓ | ✓ | ✓ | ✓ | ✓ | ✓ | ✓ | ✓ | ✓ | ✓ | ✓ | ✓ | ✓ |
|  | smoking | ✓ | ✓ | ✓ | 🗶 | ✓ | ✓ | ✓ | ✓ | 🗶 | ✓ | ✓ | ✓ | ✓ | ✓ | ✓ | ✓ | ✓ | ✓ | ✓ | ✓ | ✓ | ✓ | ✓ | ✓ | ✓ | ✓ | ✓ | ✓ | ✓ |
|  | alcohol consumption | 🗶 | ✓ | ✓ | 🗶 | ✓ | 🗶 | ✓ | 🗶 | 🗶 | ✓ | ✓ | 🗶 | ✓ | ✓ | ✓ | ✓ | ✓ | ✓ | ✓ | ✓ | ✓ | ✓ | ✓ | ✓ | ✓ | ✓ | ✓ | ✓ | ✓ |
|  | energy intake | ✓ | ✓ | ✓ | ✓ | 🗶 | 🗶 | ✓ | ✓ | ✓ | ✓ | 🗶 | ✓ | ✓ | ✓ | ✓ | ✓ | ✓ | ✓ | ✓ | ✓ | ✓ | ✓ | ✓ | ✓ | ✓ | ✓ | ✓ | ✓ | ✓ |
|  | hypertension, systolic BP | ✓ | ✓ | ✓ | ✓ | 🗶 | ✓ | ✓ | ✓ | 🗶 | ✓ | 🗶 | 🗶 | 🗶 | ✓ | ✓ | ✓ | ✓ | ✓ | ✓ | ✓ | ✓ | ✓ | ✓ | ✓ | ✓ | ✓ | ✓ | ✓ | ✓ |
| not used confounder | family history of diabetes | ✓ | 🗶 | 🗶 | 🗶 | ✓ | ✓ | ✓ | ✓ | ✓ | 🗶 | ✓ | ✓ | 🗶 | ✓ | ✓ | ✓ | ✓ | ✓ | ✓ | ✓ | 🗶 | ✓ | 🗶 | 🗶 | ✓ | ✓ | ✓ | ✓ | ✓ |
|  | waist circumferences | 🗶 | 🗶 | 🗶 | 🗶 | ✓ | 🗶 | 🗶 | 🗶 | ✓ | 🗶 | ✓ | 🗶 | 🗶 | ✓ | ✓ | ✓ | ✓ | ✓ | ✓ | ✓ | ✓ | ✓ | 🗶 | ✓ | ✓ | 🗶 | ✓ | ✓ | ✓ |
|  | ethnicity/area/dialect | ✓ | ✓ | ✓ | ✓ | 🗶 | 🗶 | ✓ | ✓ | ✓ | ✓ | 🗶 | 🗶 | 🗶 | ✓ | 🗶 | ✓ | 🗶 | 🗶 | ✓ | ✓ | ✓ | 🗶 | ✓ | ✓ | ✓ | ✓ | 🗶 | ✓ | 🗶 |
|  | cholesterol | ✓ | 🗶 | 🗶 | 🗶 | 🗶 | ✓ | 🗶 | ✓ | 🗶 | 🗶 | 🗶 | 🗶 | 🗶 | ✓ | ✓ | ✓ | ✓ | ✓ | ✓ | ✓ | ✓ | ✓ | ✓ | ✓ | 🗶 | ✓ | ✓ | ✓ | ✓ |
|  | hypercholesterolemia | 🗶 | 🗶 | 🗶 | 🗶 | 🗶 | 🗶 | ✓ | 🗶 | 🗶 | 🗶 | 🗶 | 🗶 | 🗶 | 🗶 | 🗶 | 🗶 | 🗶 | 🗶 | 🗶 | ✓ | 🗶 | 🗶 | 🗶 | 🗶 | 🗶 | 🗶 | ✓ | 🗶 | 🗶 |
|  | triglyceride | 🗶 | 🗶 | 🗶 | 🗶 | 🗶 | ✓ | 🗶 | 🗶 | 🗶 | 🗶 | 🗶 | 🗶 | 🗶 | ✓ | ✓ | ✓ | ✓ | ✓ | ✓ | ✓ | ✓ | ✓ | ✓ | ✓ | 🗶 | ✓ | ✓ | 🗶 | ✓ |
|  | marital status | 🗶 | ✓ | ✓ | 🗶 | 🗶 | 🗶 | 🗶 | 🗶 | 🗶 | ✓ | 🗶 | 🗶 | 🗶 | 🗶 | 🗶 | 🗶 | 🗶 | 🗶 | 🗶 | 🗶 | 🗶 | 🗶 | 🗶 | 🗶 | 🗶 | 🗶 | ✓ | 🗶 | 🗶 |
|  | year of interview | 🗶 | 🗶 | 🗶 | ✓ | 🗶 | 🗶 | 🗶 | 🗶 | 🗶 | 🗶 | 🗶 | 🗶 | 🗶 | - | - | - | - | - | - | - | - | - | - | - | - | - | - | - | - |
|  | blood glucose level | 🗶 | 🗶 | 🗶 | 🗶 | 🗶 | ✓ | 🗶 | 🗶 | 🗶 | 🗶 | 🗶 | 🗶 | 🗶 | - | - | - | - | - | - | - | - | - | - | - | - | - | - | - | - |
|  | fasting status | 🗶 | 🗶 | 🗶 | 🗶 | 🗶 | ✓ | 🗶 | 🗶 | 🗶 | 🗶 | 🗶 | 🗶 | 🗶 | - | - | - | - | - | - | - | - | - | - | - | - | - | - | - | - |

Checkmark indicates that a confounder was used for the published pattern or available in the respective InterConnect cohort. X indicates that it was not used or available. W indicates cohorts including only women. Cells with hyphen indicate confounders that were not checked for availability in the respective cohort because it was not considered to be relevant as it was used for only one published pattern.

**Supplemental Table 5:** Coding of covariate variables to study the association between replicated dietary patterns and incident type 2 diabetes in InterConnect.

| Study | Age (years) | Sex | Body Mass Index (kg/m²) | Smoking | Alcohol (g/day) | Energy intake (kcal/day) | Hyper-tension | Education | Physical Activity |
| --- | --- | --- | --- | --- | --- | --- | --- | --- | --- |
| AusDiab | Continuous | Men; women | Continuous | Never; former; current | Continuous | Continuous | Yes/No | Secondary; trade/vocational; tertiary | 3 categories (sedentary; insufficient; sufficient) |
| ARIC | Continuous | Men; women | Continuous | Never; former; current | Continuous | Continuous | Yes/No | Basic education or 0 years education; intermediate education; advanced education | Sport index (1-5) |
| CARDIA | Continuous | Men; women | Continuous | Never; former; current | Continuous | Continuous | Yes/No | Primary; secondary; university and beyond; none | Score (0-60): Moderate activity per week |
| CoLaus | Continuous | Men; women | Continuous | Never; former; current | Continuous | Continuous | Yes/No | University education; high school; apprenticeship; mandatory education | Total daily energy expenditure kcal/day |
| COSM/SMC | Continuous | Men (COSM); women (SMC) | Continuous | Never; former; current | Continuous | Continuous | Yes/No | Up to 9 years; 10-12 years; more than 12 years | MET*hours/d |
| ELSA-Brasil | Continuous | Men; women | Continuous | Never; former; current | Continuous | Continuous | Yes/No | Incomplete elementary school; complete elementary school; complete secondary school; university degree | minutes of MET/week |
| EPIC-InterAct | Continuous | Men; women | Continuous | Never; former; current | Continuous | Continuous | Yes/No | Primary; secondary; technical/professional; longer education | 4 categories (inactive, moderately inactive, moderately active, active) |
| Golestan | Continuous | Men; women | Continuous | Never; former; current | Never used; has used | Continuous | Yes/No | Illiterate; < 5 yrs; 6-8 yrs; 9-12 yrs; university | 3 categories (irregular non-intense; regular non-intense; irregular intense) |
| KoGES (ASAS, CAVAS) | Continuous | Men; women | Continuous | Never; former; current | Continuous | Continuous | Yes/No | Below elementary school; middle school; high school; college; university; more than a graduate school | MET/week |
| MEC | Continuous | Men; women | Continuous | Never; former; current | Continuous | Continuous | Yes/No | 3 categories (<=12y, 13-15y, 16+y) | MET/day |
| MESA | Continuous | Men; women | Continuous | Never; former; current | Continuous | Continuous | Yes/No | No schooling; grades 1-8; grades 9-11; completed high school/ged; some college but no degree; technical school certificate; associate degree; bachelor’s degree; graduate, professional school | MET-min/week |
| MTC | Continuous | Women | Continuous | Never; former; current | Continuous | Continuous | Yes/No | None; primary; special education; post-primary technician; secondary; post-secondary technician; preparatory / vocational; post-vocational technician; university; postgraduate | MET/week |
| PRHHP | Continuous | Men | Continuous | Never; former; current | Continuous | Continuous | Yes/No | None; grades 1-4; grades 5-8; high school - attended; high school - graduated; university - attended; university - graduated | hours/day |
| SUN | Continuous | Men; women | Continuous | Never; former; current | Continuous | Continuous | Yes/No | None; doctoral thesis; degree; associate's degree; master | MET-hours/week |
| WHI | Continuous | Women | Continuous | Never; former; current | Continuous | Continuous | Yes/No | 11 categories | 3 categories (low, moderate, intense) |
| Whitehall II | Continuous | Men; women | Continuous | Never; former; current | Continuous | Continuous | Yes/No | No academic qualification; ordinary level; advanced level; BA/BSc; higher degrees | MET-hours/week |

MET – metabolic equivalent of task

**Supplemental Table 6** Pooled findings of federated random effect meta-analyses to test for the association between the simplified healthy and unhealthy dietary pattern variables (per one standard deviation) (cut-off factor loadings > 0.4) and incident type 2 diabetes across InterConnect cohorts

| Healthy DP variables | outcome definition | IRR^a^ [95% CI] | I² | Tau² | p-value |
| --- | --- | --- | --- | --- | --- |
| HDP1 | primary | 1.003 [0.970 – 1.038] | 83% | 0.004 | <0.01 |
|  | secondary | 1.000 [0.975 – 1.026] | 76% | 0.003 | <0.01 |
| HDP2 | primary | n.a. | n.a. | n.a. | n.a. |
|  | secondary | n.a. | n.a. | n.a. | n.a. |
| HDP3 | primary | 0.998 [0.967 – 1.029] | 80% | 0.003 | <0.01 |
|  | secondary | 0.996 [0.971 – 1.022] | 78% | 0.003 | <0.01 |
| HDP4 | primary | 1.018 [0.985 – 1.052] | 81% | 0.004 | <0.01 |
|  | secondary | 1.009 [0.983 – 1.035] | 76% | 0.003 | <0.01 |
| HDP5 | primary | 1.012 [0.980 – 1.045] | 81% | 0.004 | <0.01 |
|  | secondary | 1.009 [0.985 – 1.034] | 74% | 0.002 | <0.01 |
| HDP6 | primary | 1.000 [0.967 – 1.034] | 82% | 0.004 | <0.01 |
|  | secondary | 0.996 [0.970 – 1.021] | 75% | 0.003 | <0.01 |
| Unhealthy DP variables |  |  |  |  |  |
| UDP1 | primary | 1.027 [0.982 – 1.074] | 80% | 0.007 | <0.01 |
|  | secondary | 1.021 [0.982 – 1.061] | 81% | 0.007 | <0.01 |
| UDP2 | primary | 0.972 [0.938 – 1.007] | 76% | 0.004 | <0.01 |
|  | secondary | 0.972 [0.944 – 1.000] | 73% | 0.003 | <0.01 |
| UDP3 | primary | **1.032 [1.016 – 1.049]** | 29% | <0.001 | <0.11 |
|  | secondary | **1.021 [1.001 – 1.042]** | 63% | 0.001 | <0.01 |
| UDP4 | primary | n.a. | n.a. | n.a. | n.a. |
|  | secondary | n.a. | n.a. | n.a. | n.a. |
| UDP5 | primary | **1.047 [1.018 – 1.078]** | 69% | 0.002 | <0.01 |
|  | secondary | 1.028 [0.999 – 1.057] | 76% | 0.003 | <0.01 |
| UDP6 | primary | **1.031 [1.006 – 1.055]** | 69% | 0.002 | <0.01 |
|  | secondary | 1.020 [0.998 – 1.041] | 70% | 0.002 | <0.01 |
| UDP7 | primary | n.a. | n.a. | n.a. | n.a. |
|  | secondary | n.a. | n.a. | n.a. | n.a. |

IRR - incidence rate ratios; I^2^ - inconsistency; n.a. – not applicable; UDP - unhealthy dietary pattern.

**Supplemental Table 7** Sensitivity analysis for the association of the unhealthy dietary pattern 3 (per one standard deviation) and incident type 2 diabetes mellitus using both outcome definitions.

|  | IRR^a^ | change in IRR [%] | I² | Tau² | p-value |
| --- | --- | --- | --- | --- | --- |
| Primary outcome definition |  |  |  |  |  |
| UDP3 | **1.104 [1.059 – 1.151]** | **ref** | 85% | 0.006 | <0.01 |
| UDP3 without rice | 1.109 [1.072 – 1.147] | +0.45 | 76% | 0.004 | <0.01 |
| UDP3 without fries | 1.104 [1.059 – 1.151] | 0 | 85% | 0.007 | <0.01 |
| UDP3 without fish | 1.097 [1.056 – 1.140] | -0.64 | 83% | 0.005 | <0.01 |
| UDP3 without red meat | 1.094 [1.052 – 1.139] | -0.91 | 85% | 0.006 | <0.01 |
| UDP3 without poultry | 1.093 [1.047 – 1.143] | -1.01 | 87% | 0.007 | <0.01 |
| UDP3 without eggs | 1.092 [1.050 – 1.136] | -1.10 | 84% | 0.006 | <0.01 |
| UDP3 without proc meat | 1.086 [1.043 – 1.130] | -1.66 | 85% | 0.006 | <0.01 |
| UDP3 without refined grain | 1.054 [1.009 – 1.101] | -4.74 | 89% | 0.007 | <0.01 |
| Secondary outcome definition |  |  |  |  |  |
| UDP3 | 1.094 [1.056 – 1.133] | **ref** | 84% | 0.006 | <0.01 |
| UDP3 without rice | 1.096 [1.065 – 1.129] | +0.18 | 76% | 0.004 | <0.01 |
| UDP3 without fries | 1.092 [1.054 – 1.131] | -0.18 | 84% | 0.006 | <0.01 |
| UDP3 without fish | 1.088 [1.052 – 1.125] | -0.60 | 83% | 0.005 | <0.01 |
| UDP3 without red meat | 1.088 [1.053 – 1.125] | -0.55 | 83% | 0.005 | <0.01 |
| UDP3 without poultry | 1.086 [1.045 – 1.128] | -0.74 | 87% | 0.007 | <0.01 |
| UDP3 without eggs | 1.082 [1.046 – 1.119] | -1.11 | 83% | 0.005 | <0.01 |
| UDP3 without proc meat | 1.079 [1.044 – 1.116] | -1.39 | 84% | 0.005 | <0.01 |
| UDP3 without refined grain | 1.047 [1.010 – 1.086] | -4.49 | 88% | 0.007 | <0.01 |

^a^Association adjusted for age, sex, BMI, physical activity, education, smoking, alcohol consumption, total energy intake and hypertension. IRR - incidence rate ratios; I^2^ - inconsistency; UDP - unhealthy dietary pattern

**
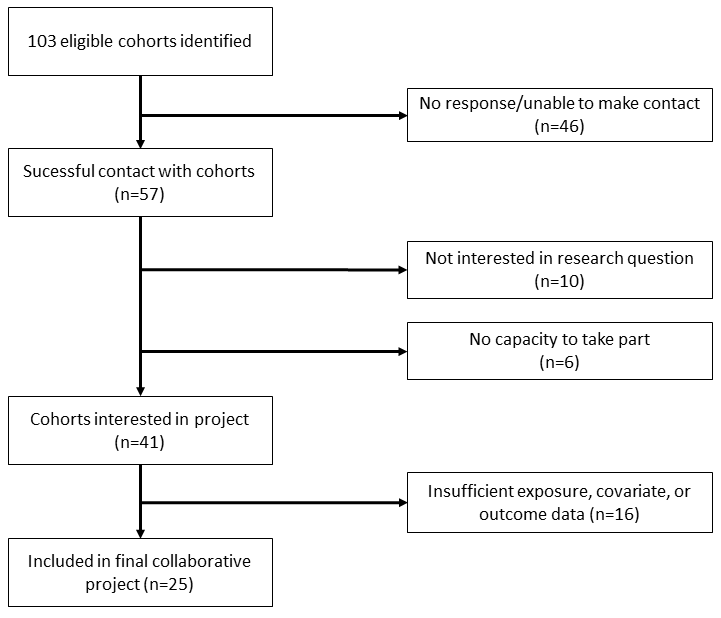
**

**Supplemental Figure 1:** Recruitment of cohorts to study the association between dietary patterns and incident type 2 diabetes in InterConnect.


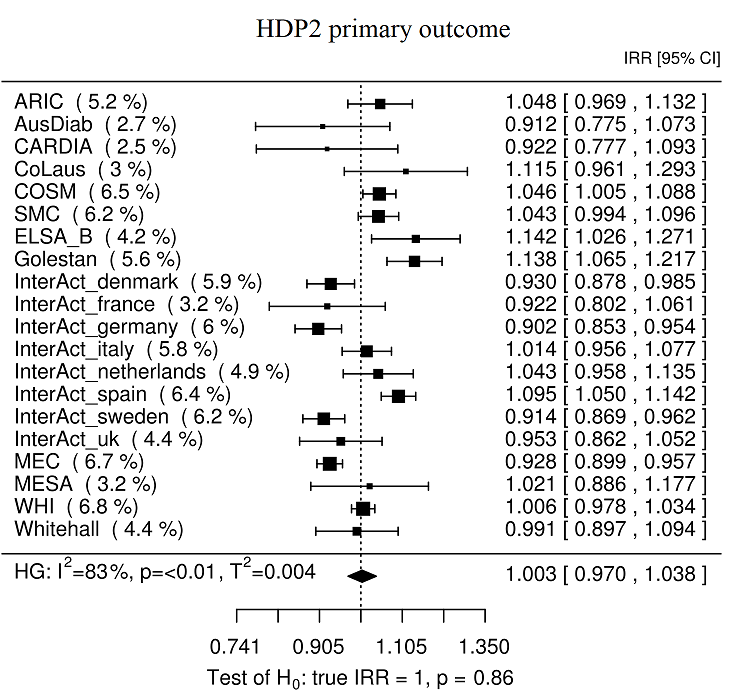

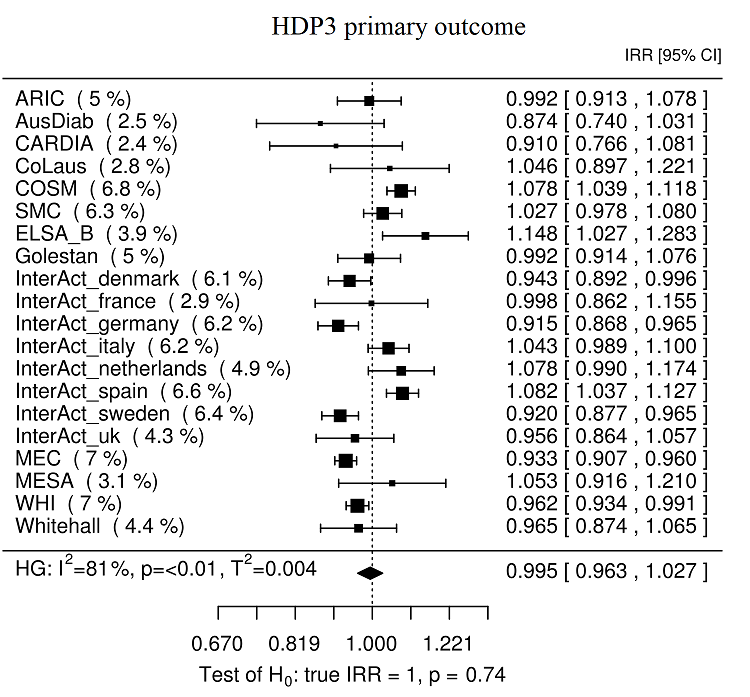

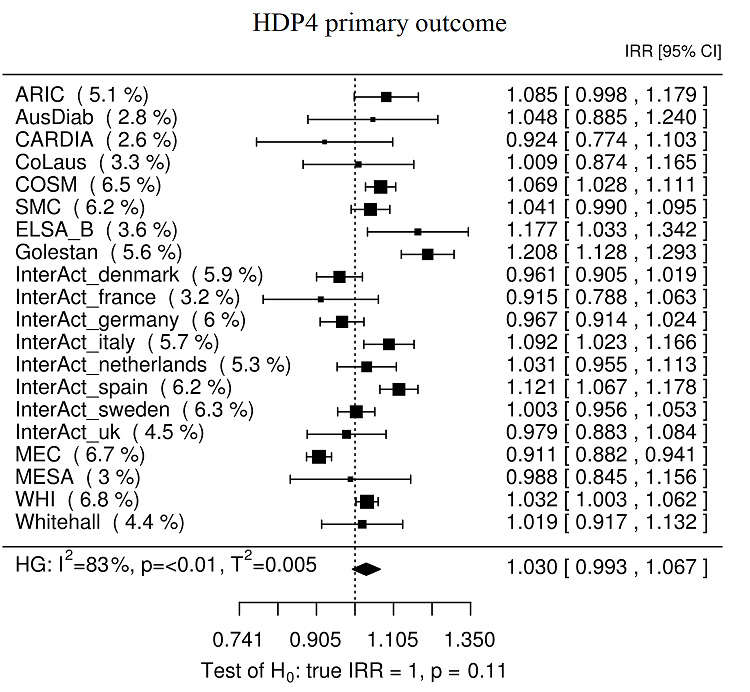

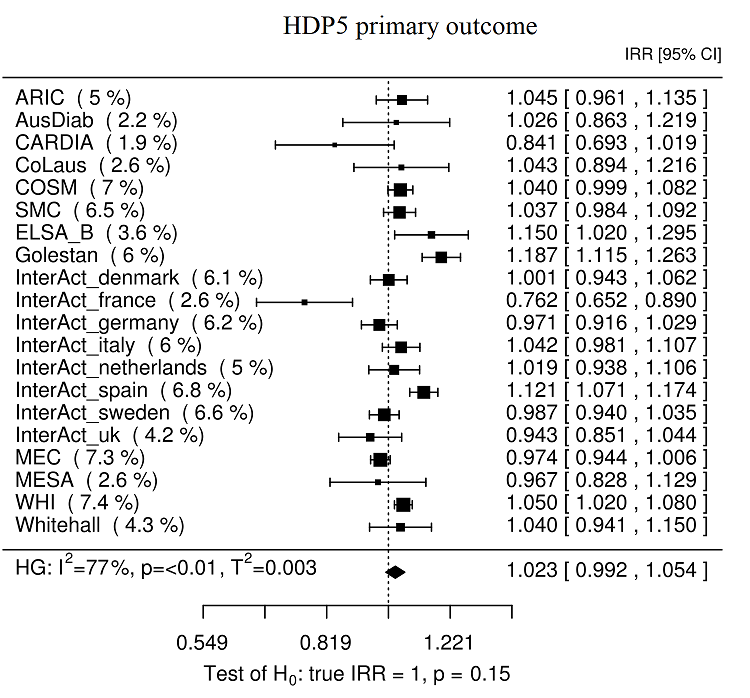

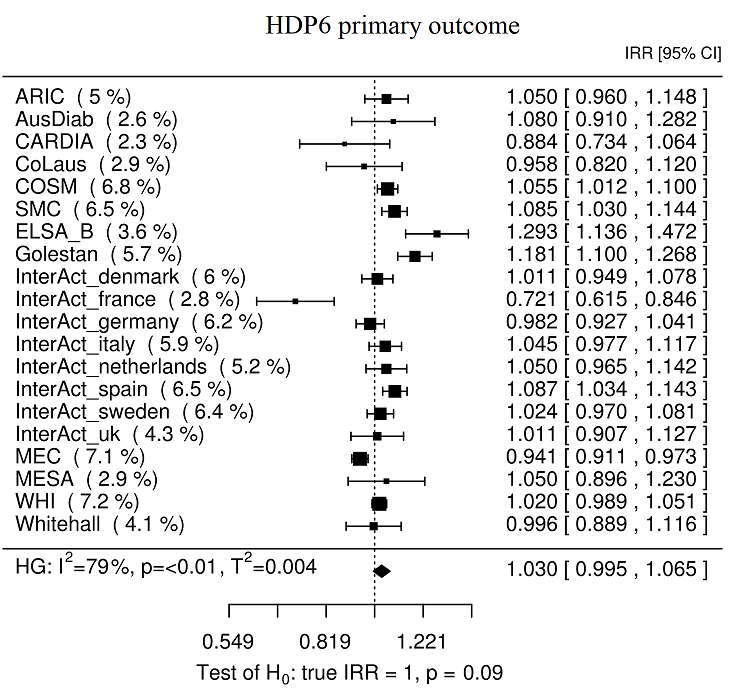

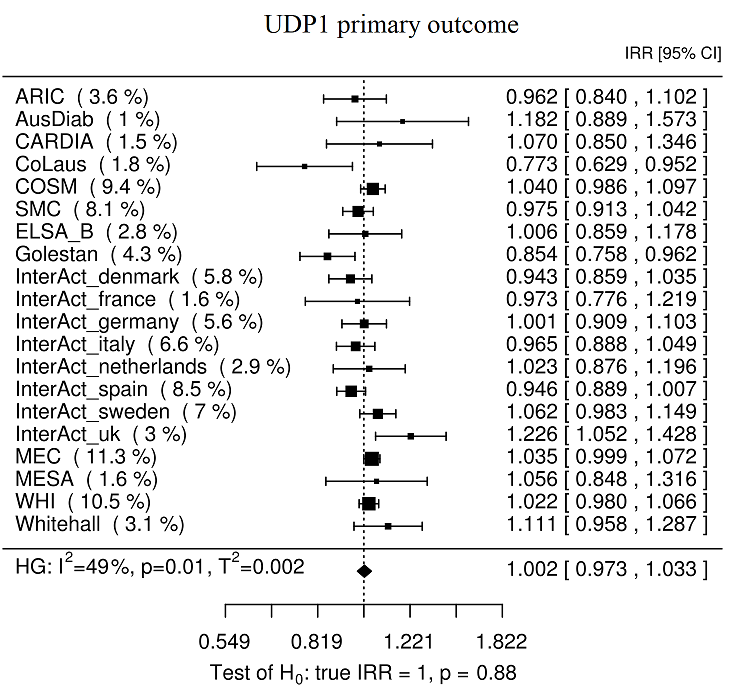


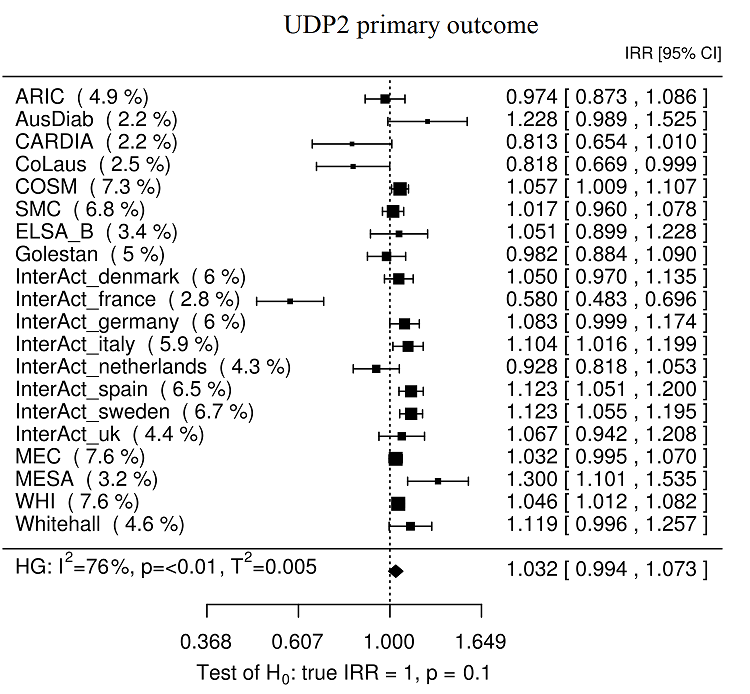


**Supplemental Figure 2:** Incidence rate ratios and 95% confidence intervals for the association between replicated dietary pattern variables and incident type 2 diabetes. Shown are results for the primary outcome definition and harmonized food groups with published factor loadings ≥ 0.2. Associations are adjusted for age, sex, BMI, physical activity, education, smoking, alcohol consumption, total energy intake and hypertension. Abbreviations: CI, confidence intervals; IRR, incidence rate ratios; HG, heterogeneity.

**
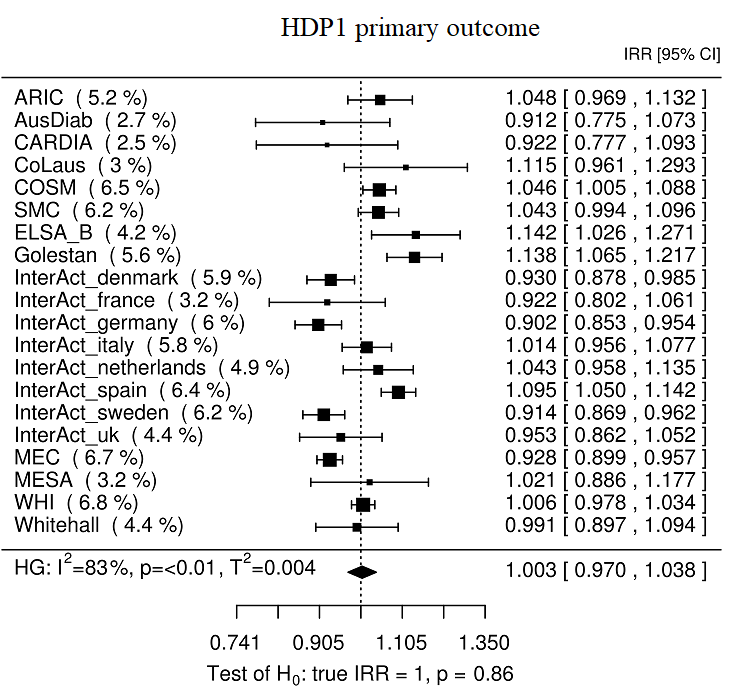

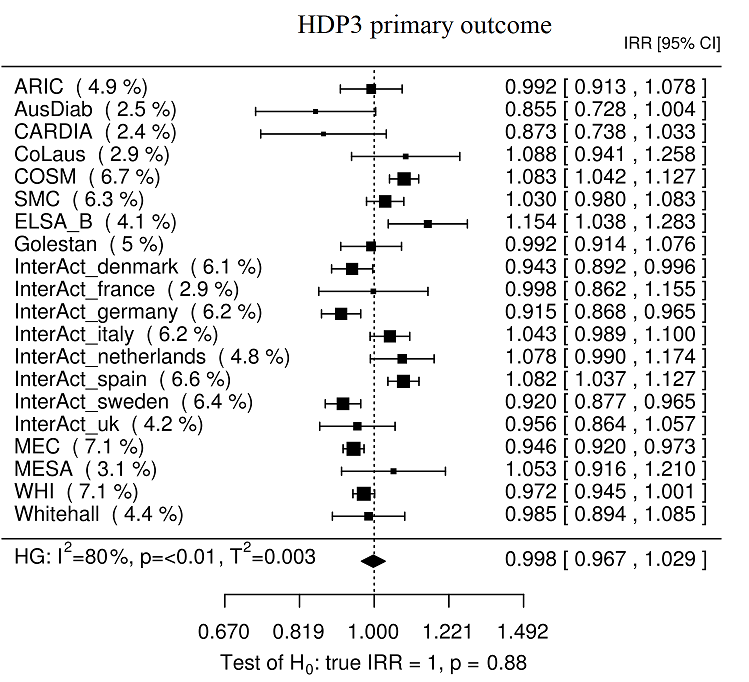

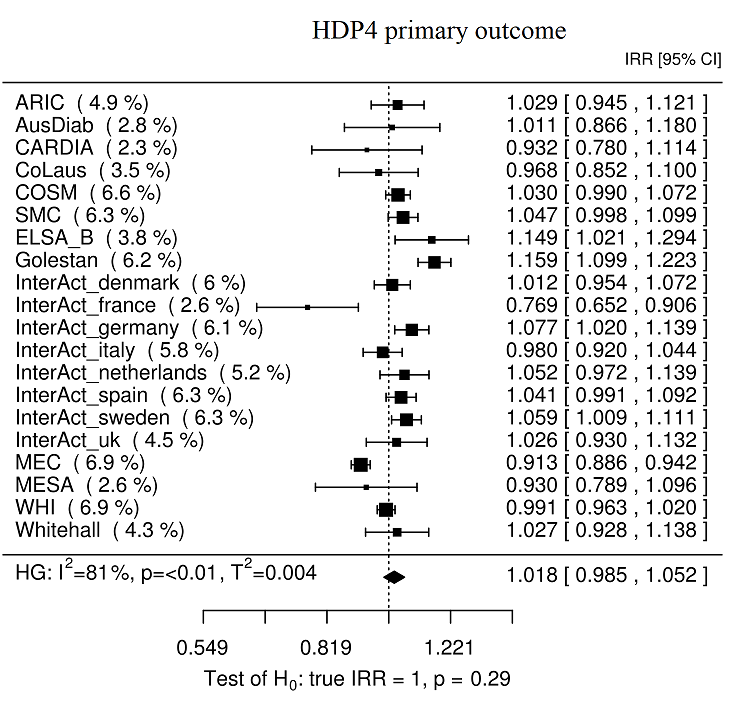

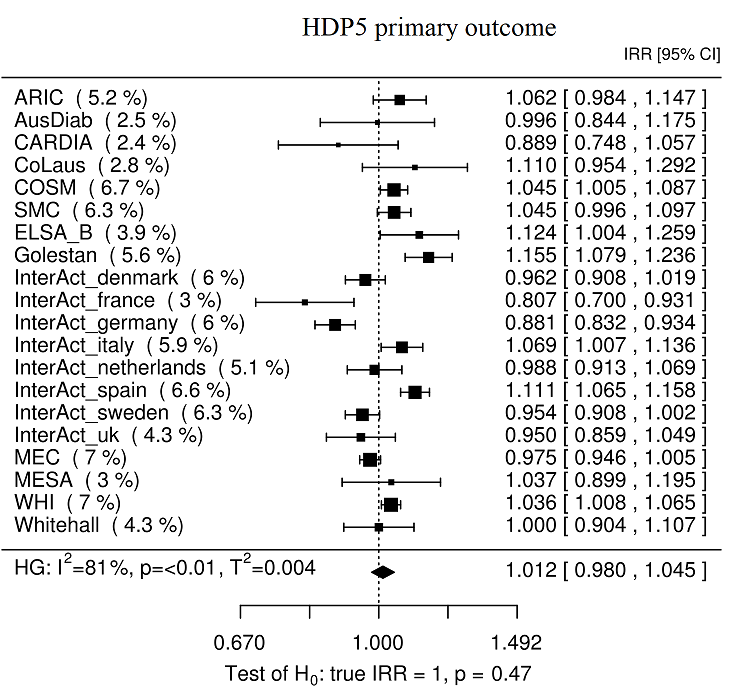

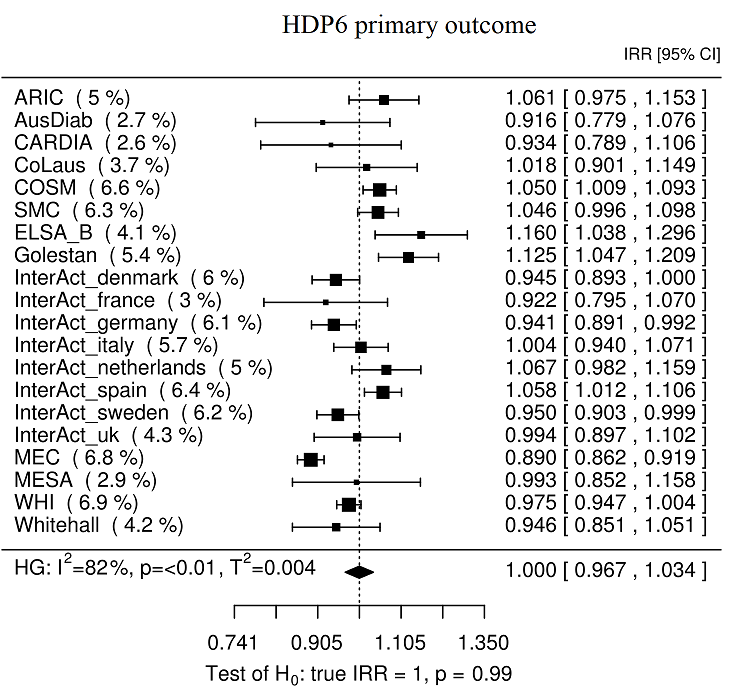

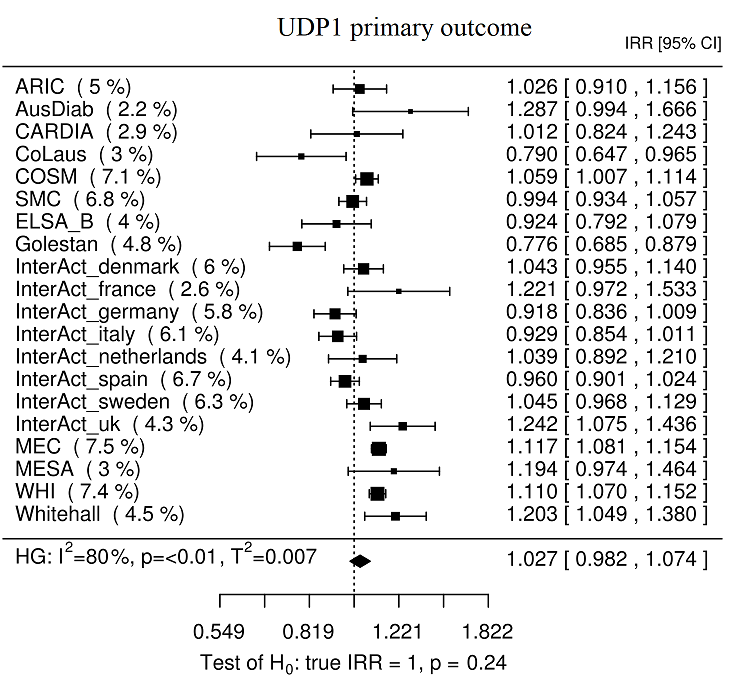

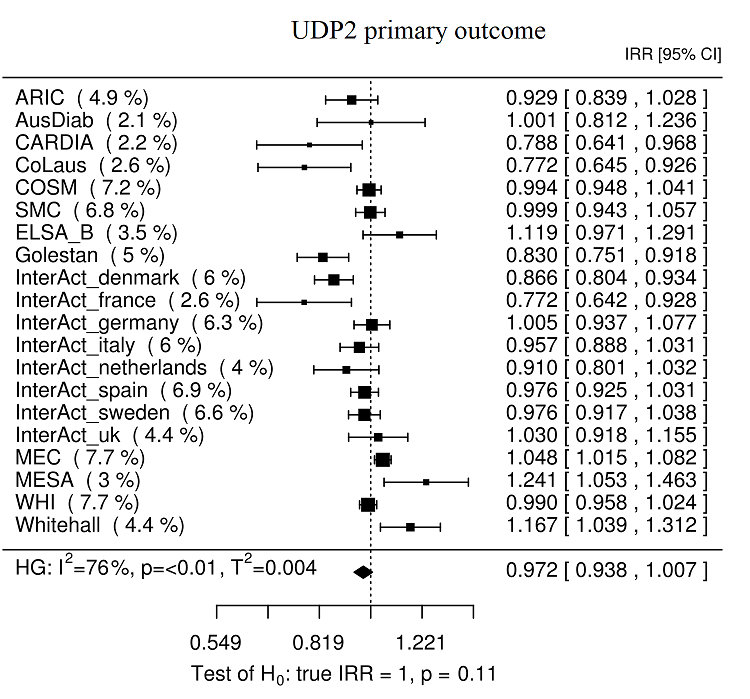

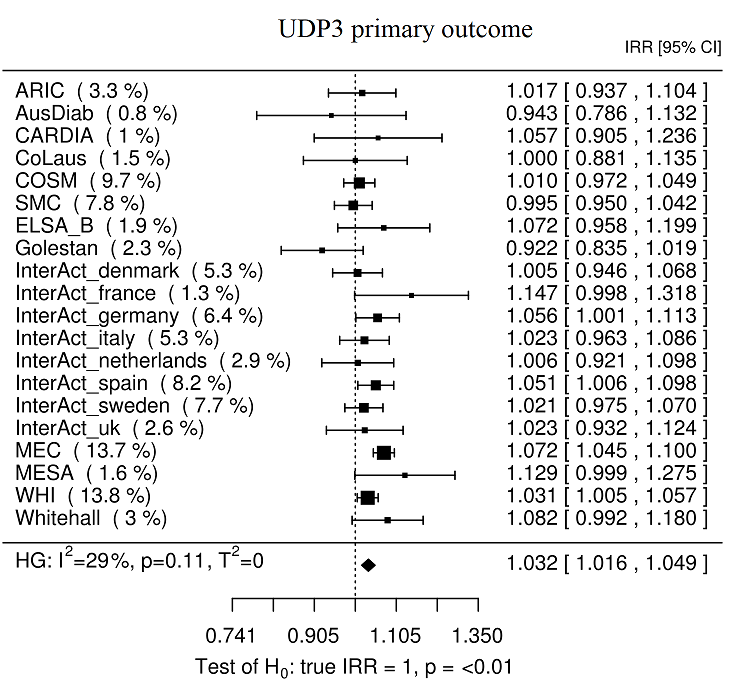

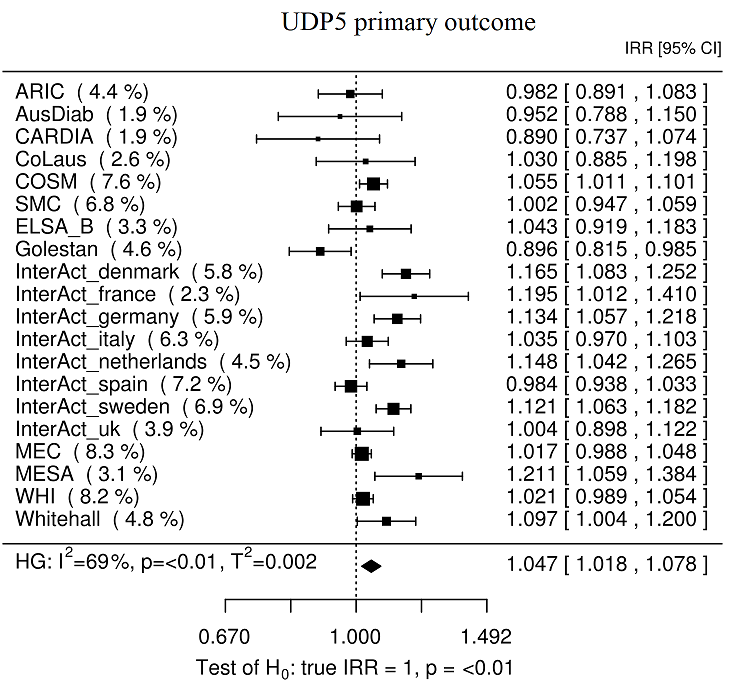

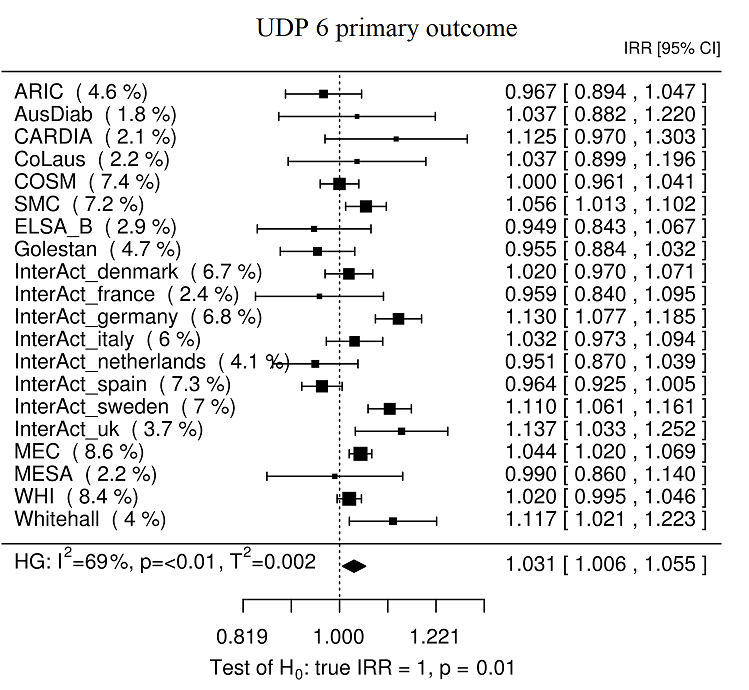
 Supplemental Figure 3:** Incidence rate ratios and 95% confidence intervals for the association between replicated dietary pattern variables and incident type 2 diabetes. Shown are results for the primary outcome definition and harmonized food groups with published factor loadings ≥0.4. Associations are adjusted for age, sex, BMI, physical activity, education, smoking, alcohol consumption, total energy intake and hypertension. Abbreviations: CI, confidence intervals; IRR, incidence rate ratios; HG, heterogeneity.

**
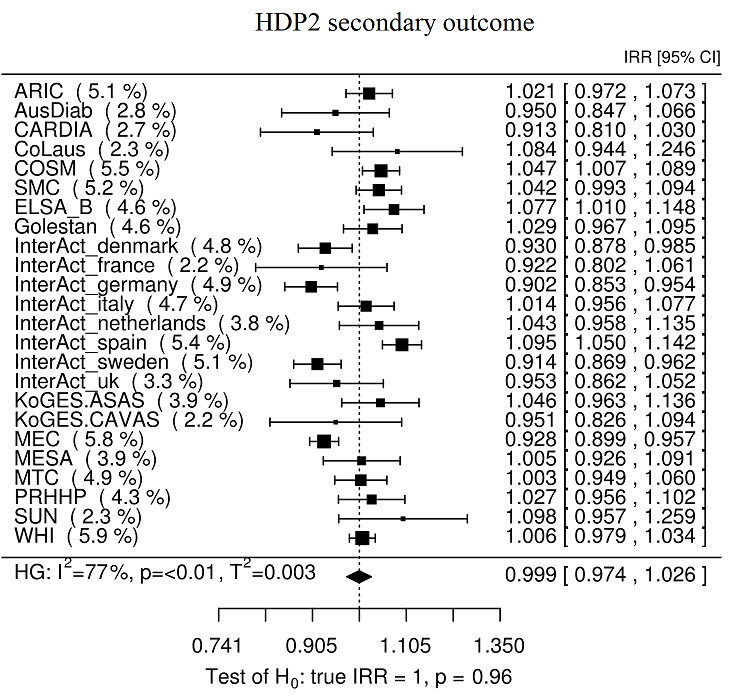

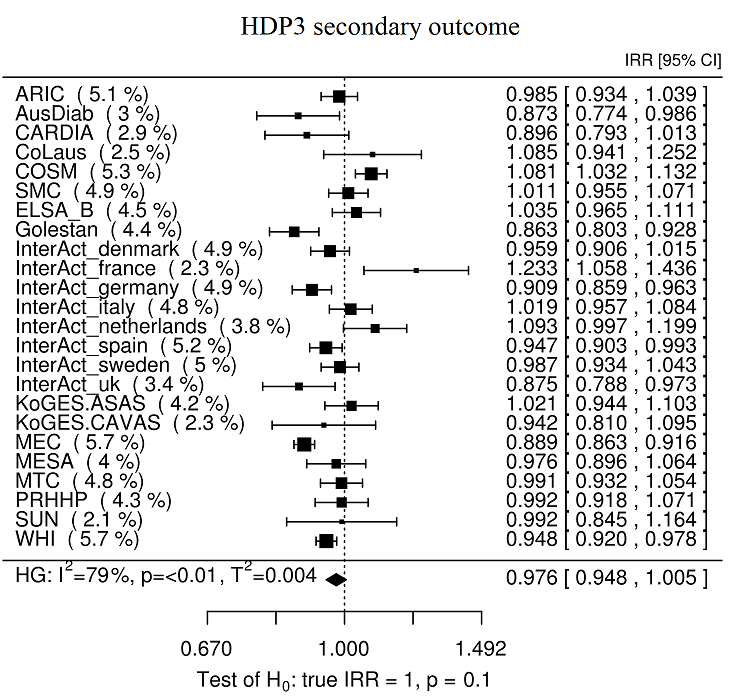

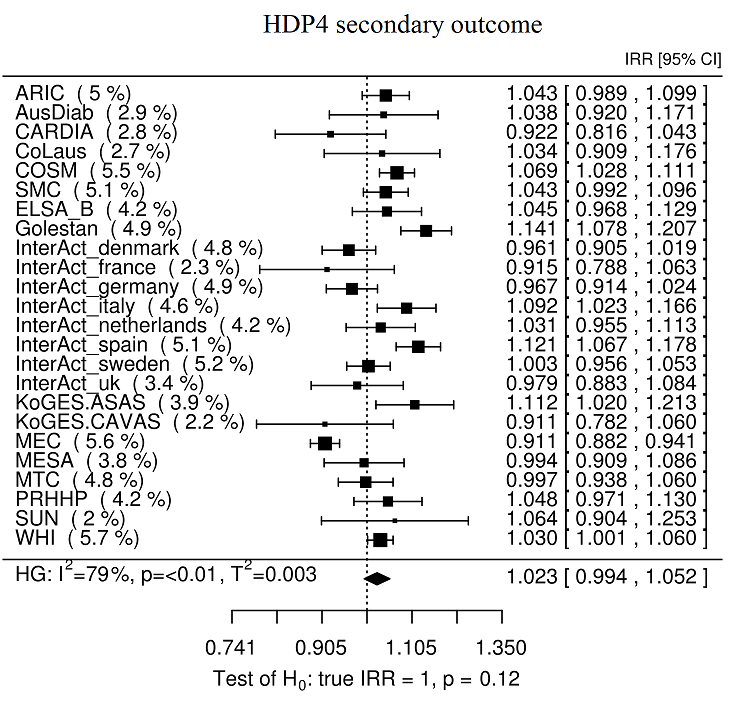

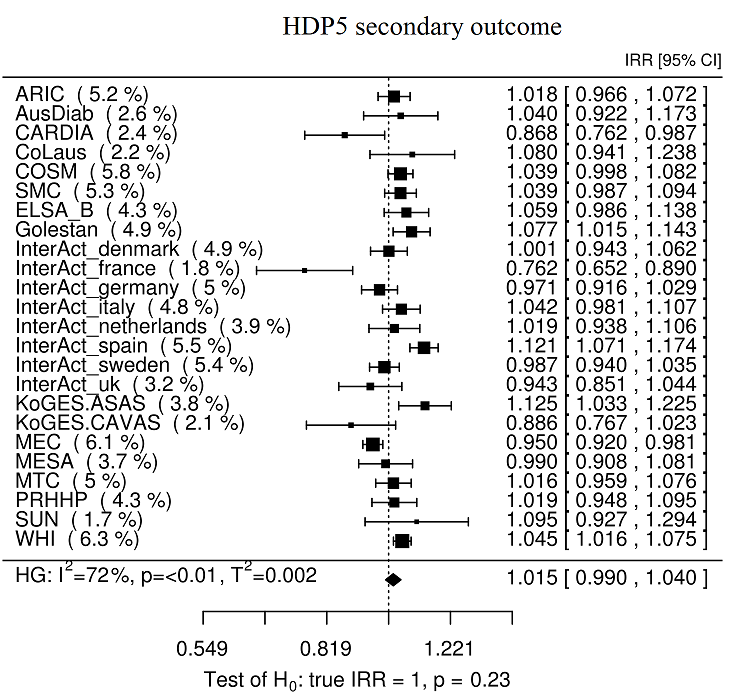

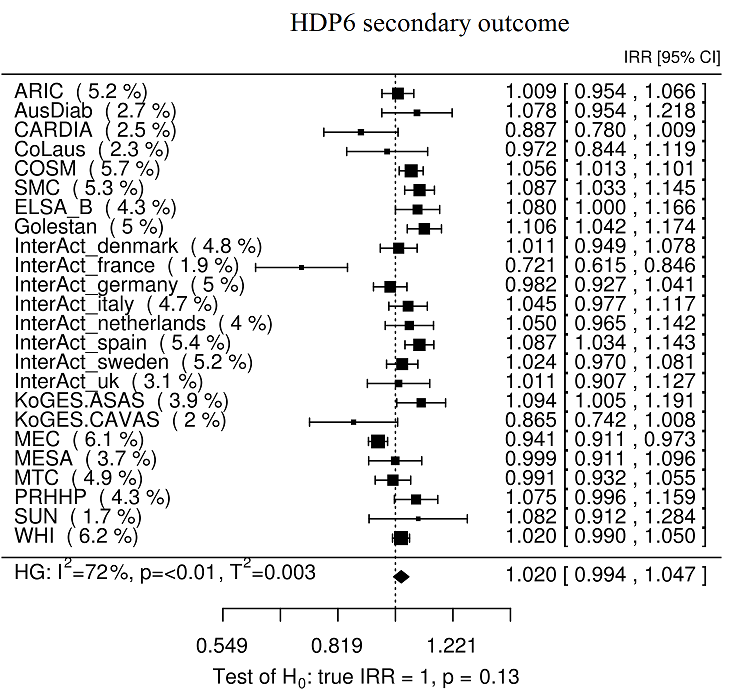

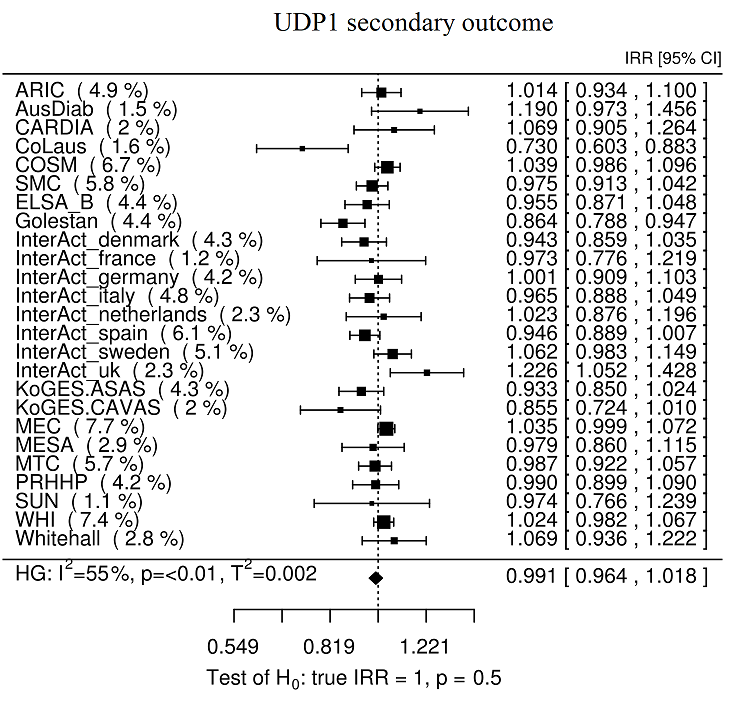
**

**
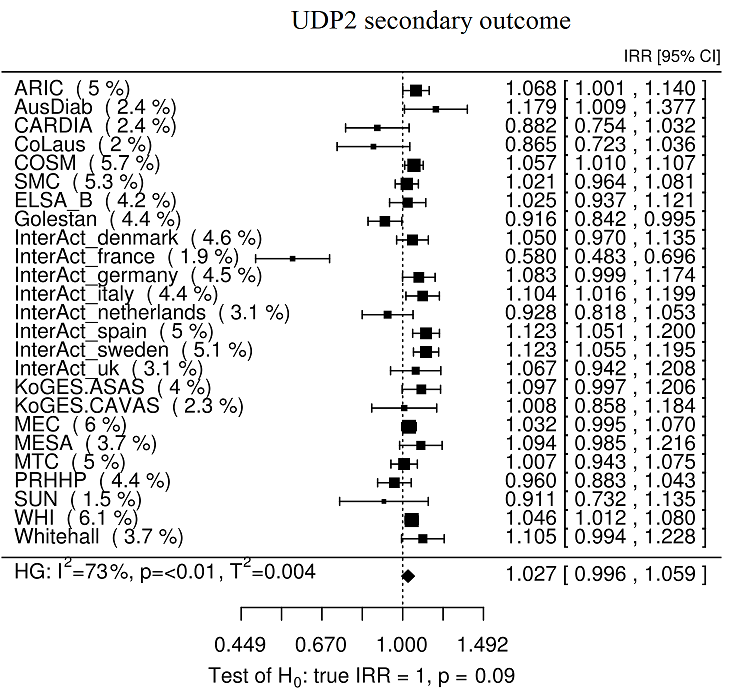
**

**Supplemental Figure 4:** Incidence rate ratios and 95% confidence intervals for the association between replicated dietary pattern variables and incident type 2 diabetes. Shown are results for the secondary outcome definition and harmonized food groups with published factor loadings ≥0.2. Associations are adjusted for age, sex, BMI, physical activity, education, smoking, alcohol consumption, total energy intake and hypertension. Abbreviations: CI, confidence intervals; IRR, incidence rate ratios; HG, heterogeneity.

**
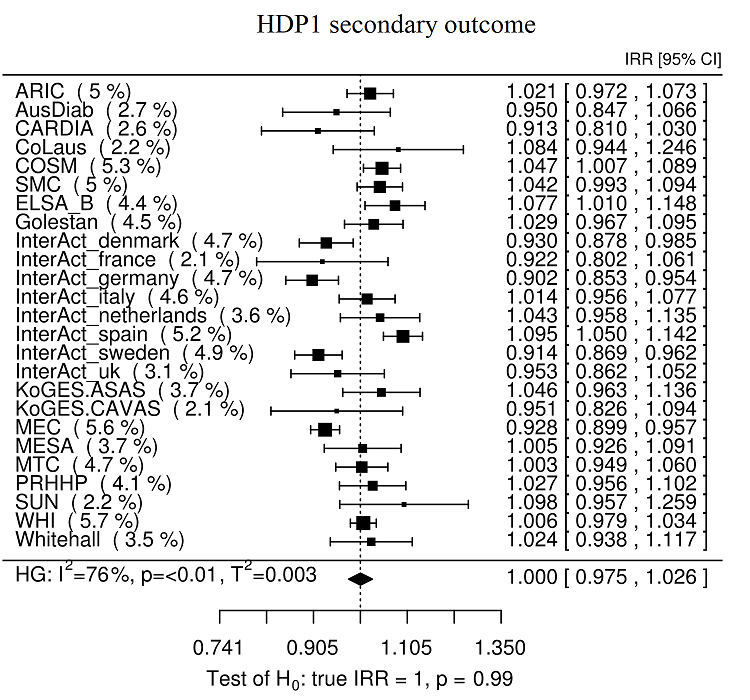

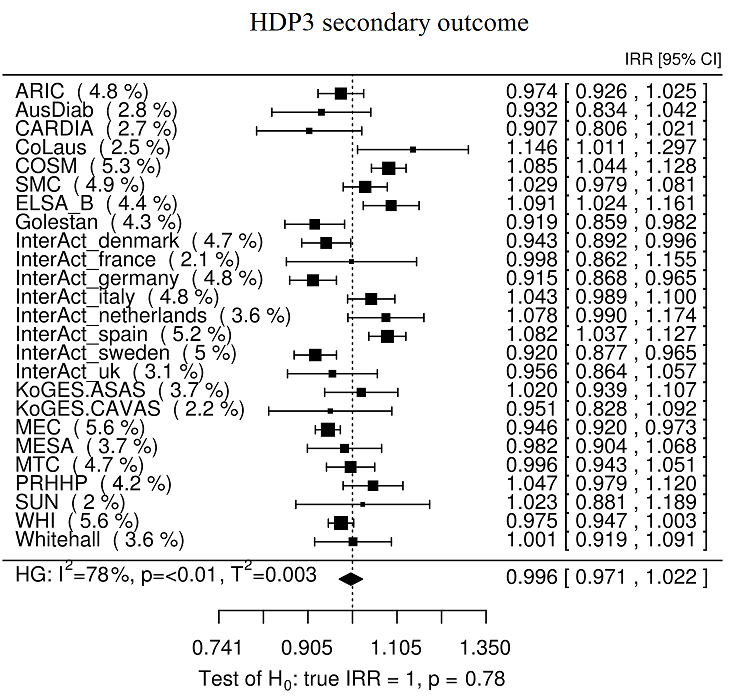

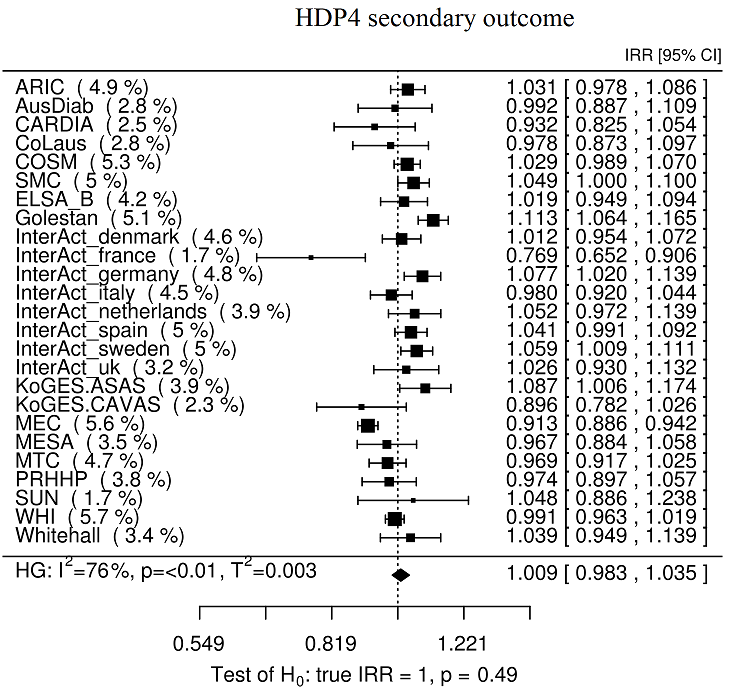

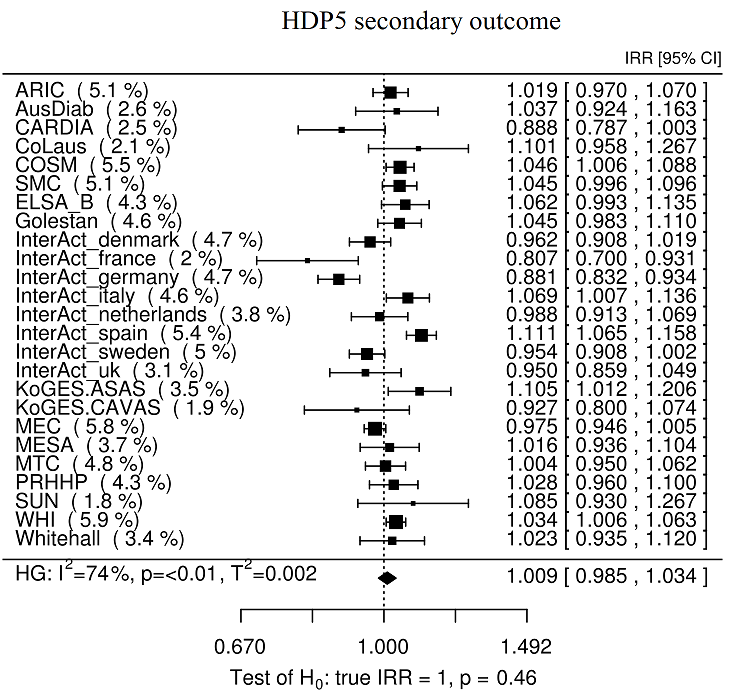

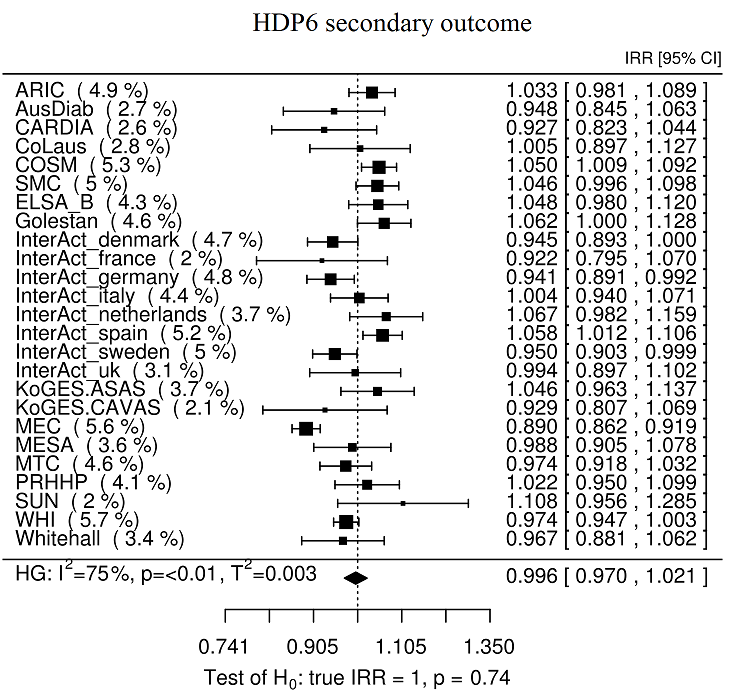

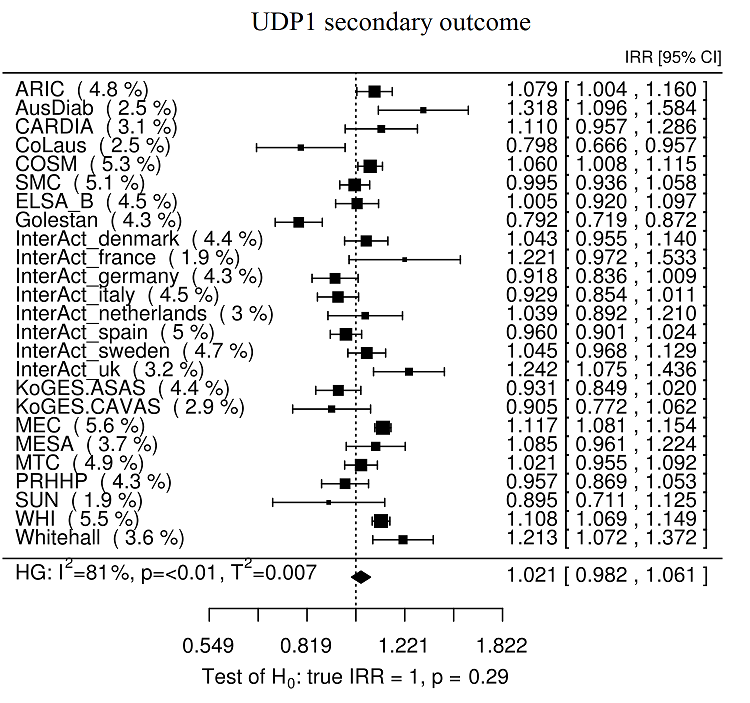

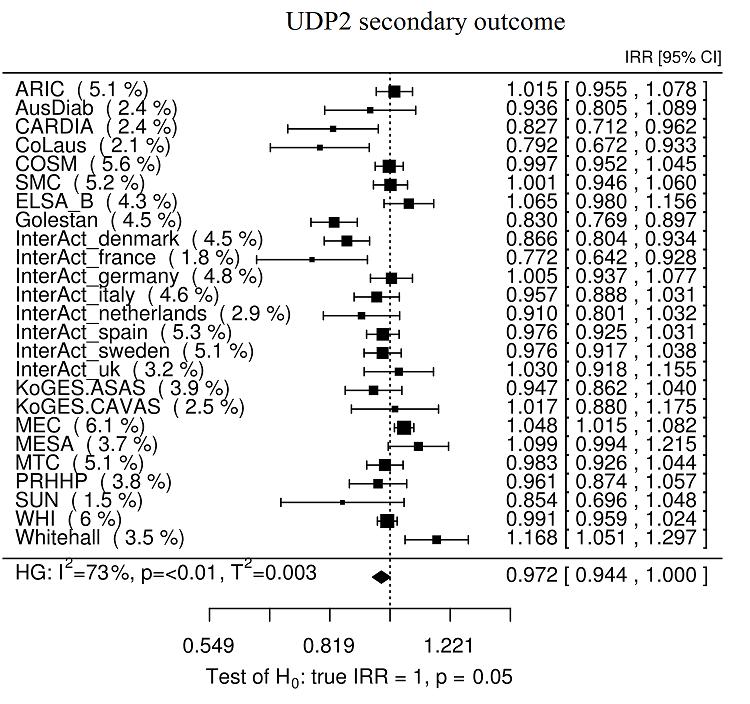

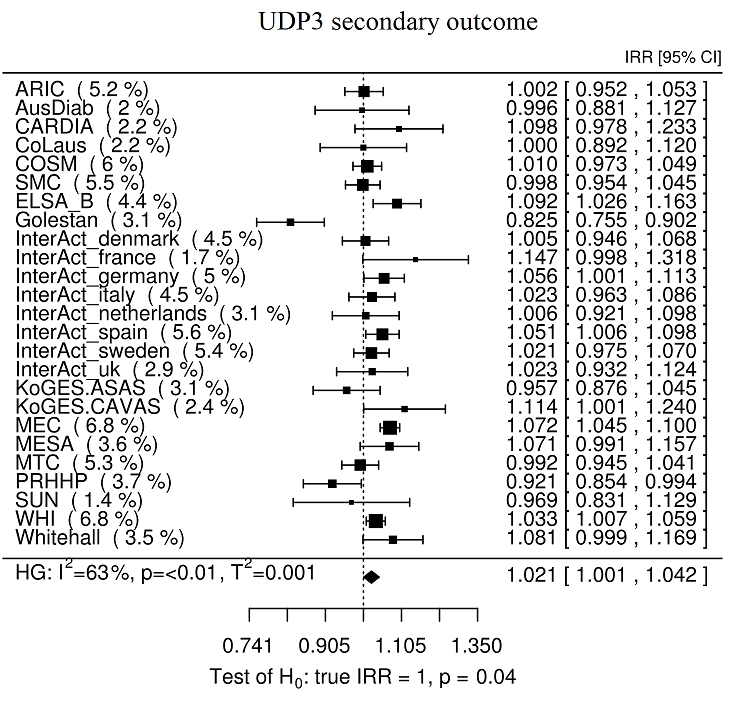

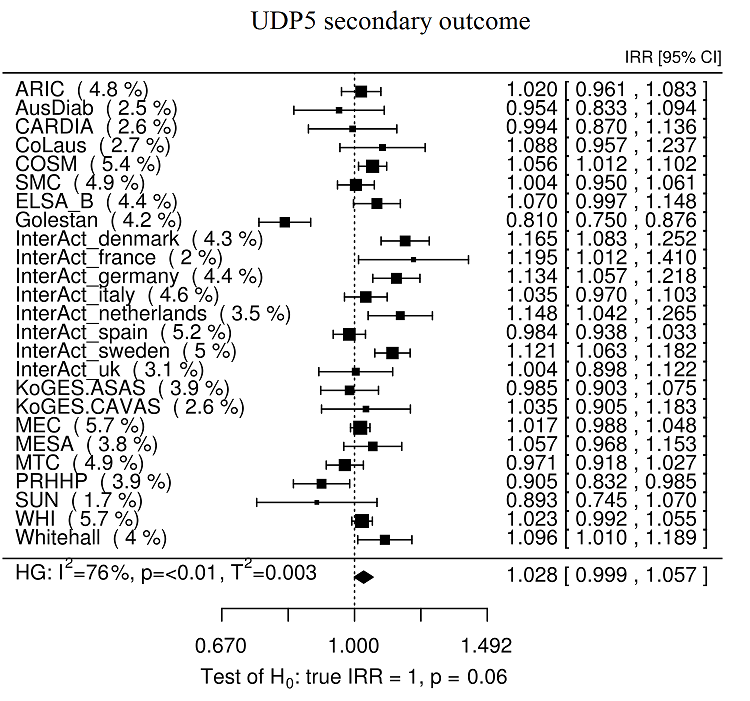

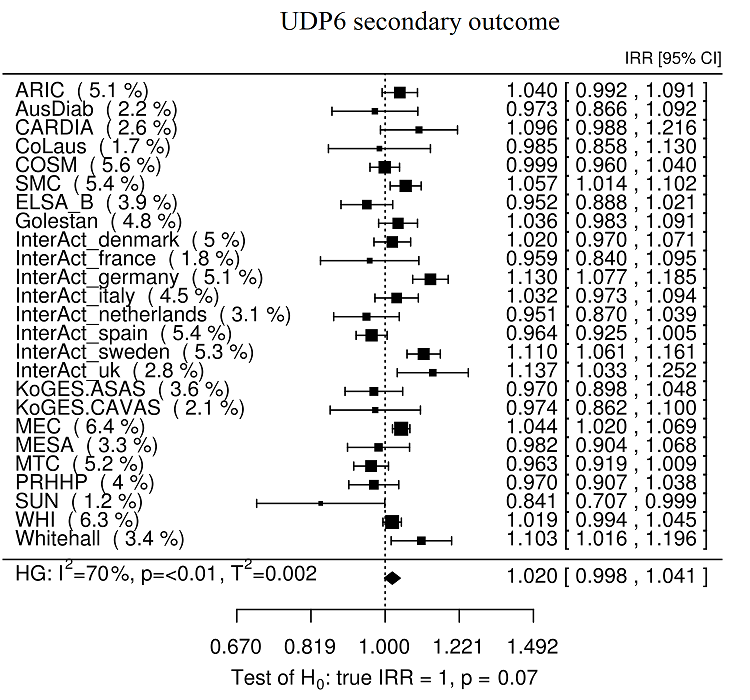
 Supplemental Figure 5:** Incidence rate ratios and 95% confidence intervals for the association between replicated dietary pattern variables and incident type 2 diabetes. Shown are results for the secondary outcome definition and harmonized food groups with published factor loadings ≥0.4. Associations are adjusted for age, sex, BMI, physical activity, education, smoking, alcohol consumption, total energy intake and hypertension. Abbreviations: CI, confidence intervals; IRR, incidence rate ratios; HG, heterogeneity.


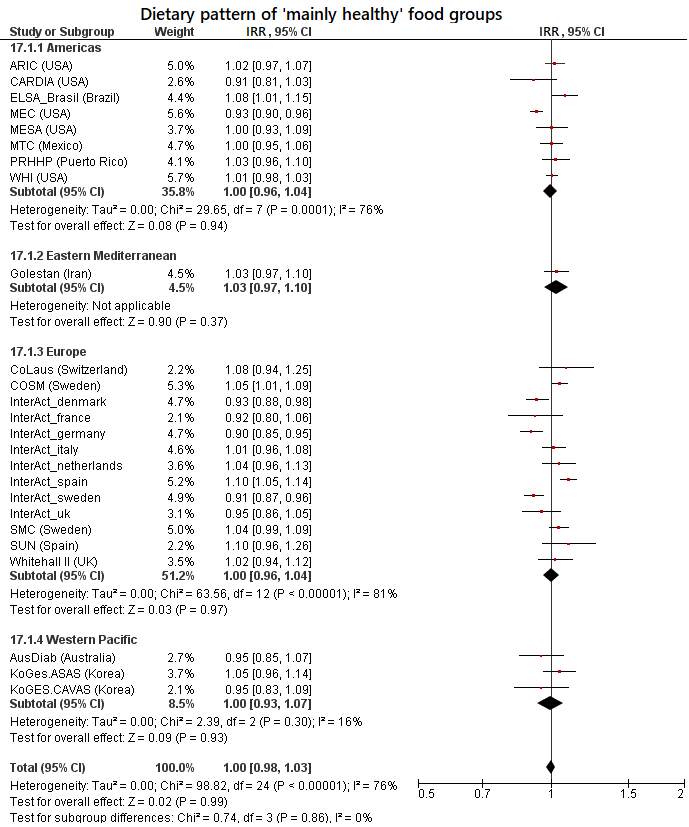

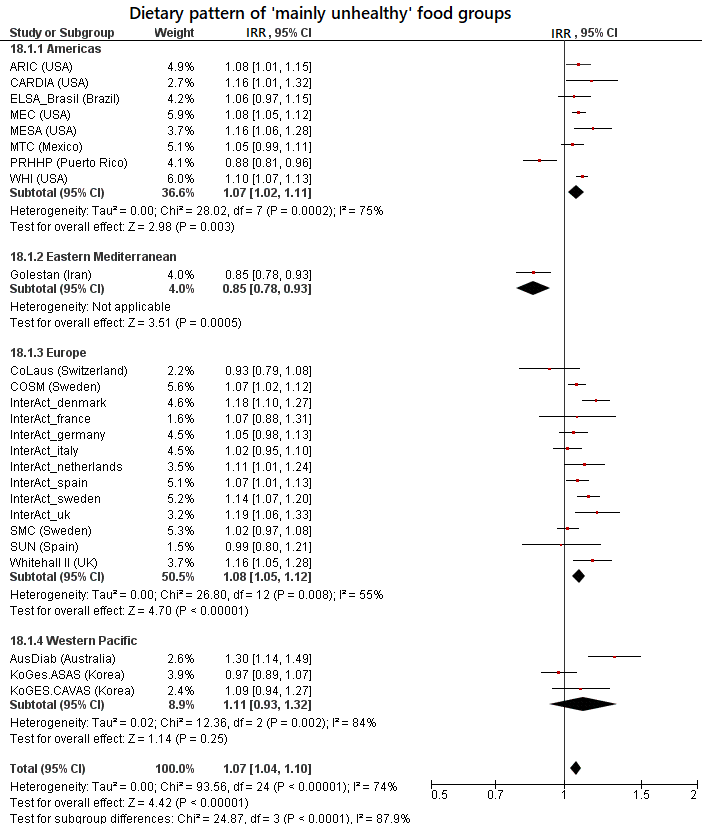


**Supplemental Figure 6** Incidence rate ratios and 95% confidence intervals for the association between the dietary patterns of “mainly healthy” and “mainly unhealthy” food groups and incident type 2 diabetes using the secondary outcome. Associations are shown by subgroups of region and adjusted for age, sex, BMI, physical activity, education, smoking, alcohol consumption, total energy intake and hypertension. Abbreviations: CI, confidence intervals; IRR, incidence rate ratios;
